# *C. elegans* Eats Its Own Intestine to Make Yolk Leading to Multiple Senescent Pathologies

**DOI:** 10.1016/j.cub.2018.06.035

**Published:** 2018-08-20

**Authors:** Marina Ezcurra, Alexandre Benedetto, Thanet Sornda, Ann F. Gilliat, Catherine Au, Qifeng Zhang, Sophie van Schelt, Alexandra L. Petrache, Hongyuan Wang, Yila de la Guardia, Shoshana Bar-Nun, Eleanor Tyler, Michael J. Wakelam, David Gems

**Affiliations:** 1 Institute of Healthy Ageing and Department of Genetics, Evolution and Environment, University College London, London WC1E 6BT, UK; 2 School of Biological and Chemical Sciences, Queen Mary University of London, London E1 4NS, UK; 3 Division of Biomedical and Life Sciences, Faculty of Health and Medicine, Lancaster University, Lancaster LA1 4YW, UK; 4 Department of Biochemistry, Faculty of Medical Science, Naresuan University, Phitsanulok 65000, Thailand; 5 The Babraham Institute, Babraham Research Campus, Cambridge CB22 3AT, UK; 6 The George S. Wise Faculty of Life Sciences, Tel-Aviv University, Tel-Aviv, Israel

**Keywords:** aging, atrophy, autophagy, *C. elegans*, intestine, insulin/IGF-1 signaling, pathology, steatosis, vitellogenin, yolk

## Abstract

Aging (senescence) is characterized by the development of numerous pathologies, some of which limit lifespan. Key to understanding aging is discovery of the mechanisms (etiologies) that cause senescent pathology. In *C. elegans*, a major senescent pathology of unknown etiology is atrophy of its principal metabolic organ, the intestine. Here we identify a cause of not only this pathology but also of yolky lipid accumulation and redistribution (a form of senescent obesity): autophagy-mediated conversion of intestinal biomass into yolk. Inhibiting intestinal autophagy or vitellogenesis rescues both visceral pathologies and can also extend lifespan. This defines a disease syndrome leading to multimorbidity and contributing to late-life mortality. Activation of gut-to-yolk biomass conversion by insulin/IGF-1 signaling (IIS) promotes reproduction and senescence. This illustrates how major, IIS-promoted senescent pathologies in *C. elegans* can originate not from damage accumulation but from direct effects of futile, continued action of a wild-type biological program (vitellogenesis).

## Highlights

• *C. elegans* consume their own intestine to synthesize yolk and promote reproduction
• This causes diseases of aging, including atrophy of the intestine and yolk steatosis
• Intestinal senescence in *C. elegans* is promoted by autophagy
• Here destructive run-on of wild-type biological programs causes senescent pathologies

## Graphical Abstract

**Figure:**
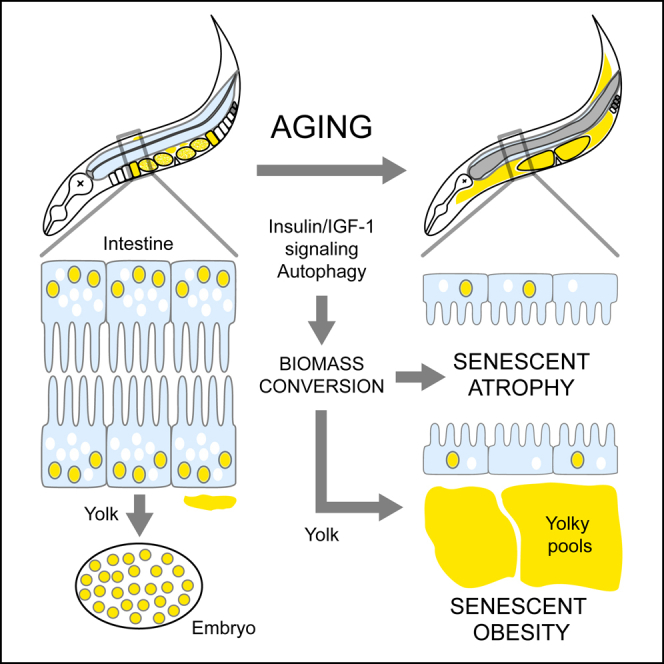

## Teaser

Ezcurra, Benedetto et al. identify a mechanism causing aging in *C. elegans*: autophagy-dependent gut-to-yolk biomass conversion. This is activated by life-shortening insulin/IGF-1 signaling and causes diseases of aging, including intestinal atrophy and yolk steatosis. The mechanism involved fits the recently proposed hyper-function theory of aging.

## Introduction

Aging is a pervasive phenomenon across animal species and the leading cause of human morbidity and death worldwide through its associated senescent pathologies. Despite major advances in the genetics of longevity, sparked by the identification of long-lived insulin/insulin-like growth factor (IGF-)1 signaling (IIS) pathway mutants in the roundworm *Caenorhabditis elegans* [1, 2, 3], the proximate causes of aging remain unclear. One proposed cause is stochastic molecular damage, for example due to reactive oxygen species (ROS) [4], and consequent accumulation of malfunctioning biomolecules, which IIS promotes [3]. However, it now appears that much aging-associated molecular damage is more consequence than cause of senescent pathologies [5, 6, 7].

Another possible cause of aging is the continued and deleterious action (run-on, or hyper-function) in later life of wild-type genes beyond their “intended purpose” [5, 8, 9]. This follows from the evolutionary principle of antagonistic pleiotropy (AP), where natural selection can favor gene variants that enhance fitness in early life while promoting senescent pathologies in later life, because the early-life benefits to the species outweigh the late-life costs to the individual [8]. For example, IIS promotes growth and reproduction in early life, and age-related pathologies in late life [6, 10]. Conversely, IIS inhibition increases lifespan and healthspan from worms to mammals [3], but can slow development and reduce fitness.

**Figure 1:**
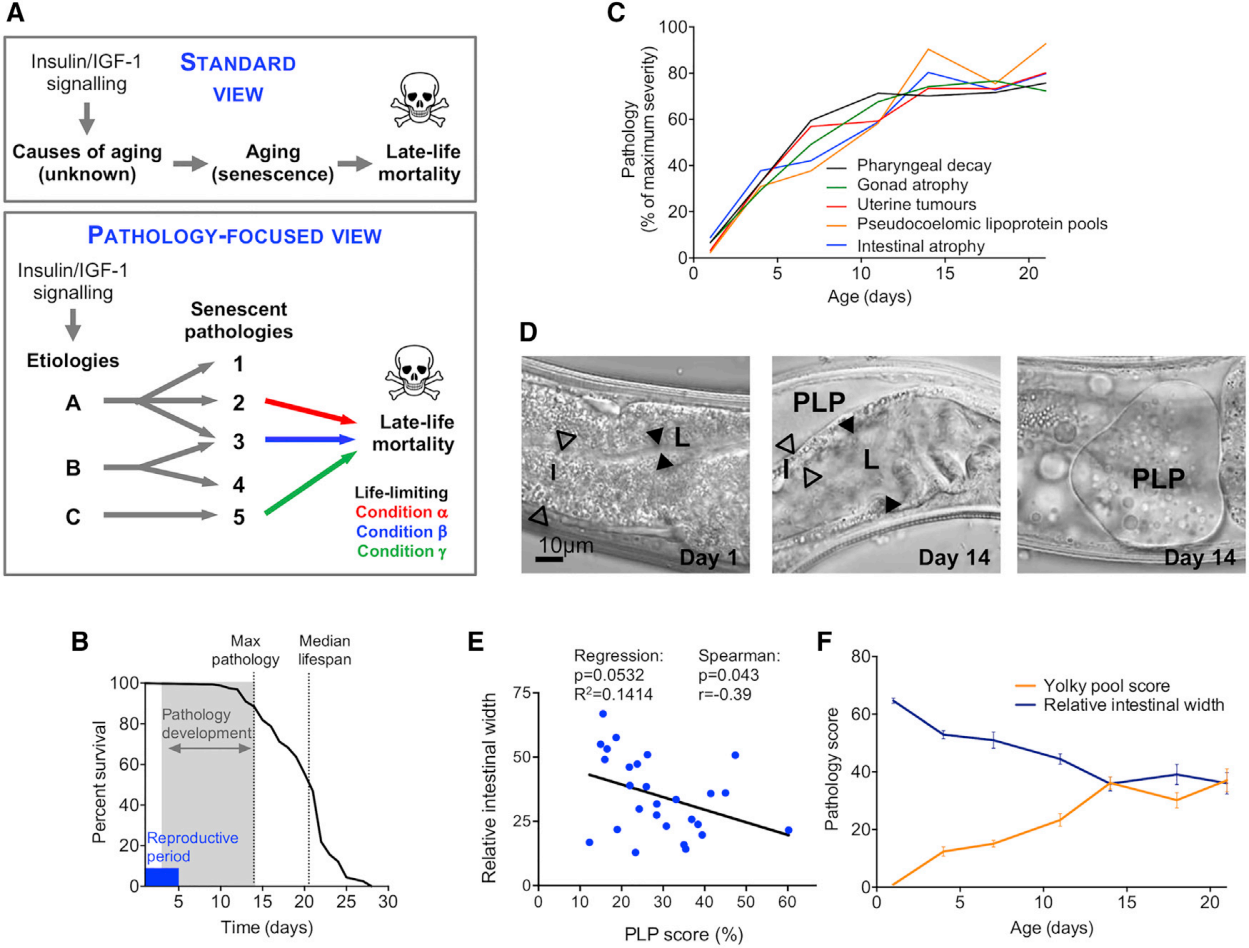
Early, Rapid, and Synchronous Development of Senescent Pathologies during *C. elegans* Adulthood (A) View of the position of senescent pathology in aging upon which this study is based. Top: lifespan is widely used as a metric of an underlying process, aging. Bottom: lifespan is additionally a function of life-limiting senescent pathologies, with differing and condition-dependent effects on late-life mortality. (B) In selfing wild-type hermaphrodites (20°C), adulthood is characterized by 4–5 days of reproduction, ∼12 days during which the gross pathologies studied here develop, and a 20-day median lifespan. (C) Many senescent pathologies develop in parallel from early adulthood, reaching maximum severity by around day 14. (D) 14-day-old hermaphrodites (center and right) exhibit an atrophied intestine (I; hollow arrowheads), an enlarged lumen (L; black arrowheads), and large pseudocoelomic lipoprotein pools (PLPs) compared to 1-day-old worms (left). (E) PLP accumulation score and intestinal atrophy correlate at the individual worm level (day 7). (F) The evolution of PLP accumulation mirrors intestinal atrophy, and their severities are strongly correlated at a population level Animals were maintained on *E. coli* OP50 at 20°C without FUDR. See also Figure S1.

The properties of long-lived mutants suggest that multiple aging pathologies can arise from common underlying mechanisms. This argues for complementing the standard lifespan genetics approaches, that have been so fruitful in invertebrate biogerontology, with the study of senescent pathologies, to enhance our understanding of aging. Hence, lifespan that has long been employed as a metric of an underlying aging process (Figure 1A, top) may also be viewed as a function of one or more life-limiting senescent pathologies. Which of the many extant pathologies limits lifespan may vary between individuals, environmental conditions, genders, and species (Figure 1A, bottom) [11, 12], confounding studies of the genetics of lifespan. A pathology-focused approach in aging model organisms can yield new insights into the primary mechanism(s) of aging and help understand how genes control lifespan.

*C. elegans* exhibits a plethora of unusually severe senescent pathologies, including degeneration of the pharynx, tumors in the uterus, atrophy of the intestine and gonad, and steatotic lipoprotein redistribution [9, 13, 14, 15, 16], thus providing ample raw material for study. Here we focus on senescent pathology in the main *C. elegans* metabolic organ, the intestine, which in addition fulfills the functions of liver and adipose tissue and is a key site of action for IIS-mediated effects on lifespan [17]. To this end, we employed a *developmental pathology* approach, quantifying the temporal evolution of gut pathology at anatomical, cellular, and molecular levels, and identified a novel pathophysiological mechanism: autophagy-dependent gut-to-yolk biomass conversion, run-on of which causes multiple visceral pathologies, including intestinal atrophy. These results are consistent with the account provided by Williams [8] and Blagosklonny [5, 9] of how antagonistic pleiotropy is enacted in terms of proximate mechanisms: destructive run-on of biological programs specified by wild-type genes.

## Results

### Major Senescent Pathologies Develop in Early to Mid-Adulthood

Various senescent pathologies have been documented in *C. elegans*, but details of their developmental timing, particularly in relation to one another, remain sparse. In humans, diseases of aging (e.g., cardiovascular, neurodegenerative, cancer) increase mainly toward the end of life. We asked: is this true of *C. elegans* too? To this end, we monitored development of uterine tumors, gonadal atrophy, deterioration of the pharynx (foregut), yolky lipoprotein pools in the body cavity (pseudocoelom), and intestinal atrophy. Wild-type (N2) hermaphrodites were aged under standard conditions (nematode growth medium [NGM] agar plates, *Escherichia coli* OP50 bacteria as a food source, 20°C) and imaged using Nomarski microscopy at intervals from day 1 to 21 (d1–d21) of adulthood, and the severity of pathologies was quantified (Figures S1A–S1E). Against expectation, senescent pathologies developed not toward the end of life but much earlier, starting at around the time of self-sperm depletion (d3–d4) and reaching peak severity at around d10–d12 of adulthood, prior to median lifespan (Figure 1B). Moreover, the various pathologies developed in relative synchrony, revealing an aging syndrome (Figure 1C). In particular, development of intestinal atrophy and pseudocoelomic lipoprotein pool (PLP) accumulation (Figure 1D) were strongly coupled in all growth conditions tested (Figure S2A), showing correlation within individuals (Figures 1E and S2B) and strikingly close temporal correlation (Figure 1F). This suggested a possible common etiology of these two pathologies, which we investigated further, beginning with a closer examination of the two pathologies individually.

### Accumulation of Pseudocoelomic Lipoprotein Pools (PLPs) as a Form of Senescent Obesity in *C. elegans*

**Figure 2:**
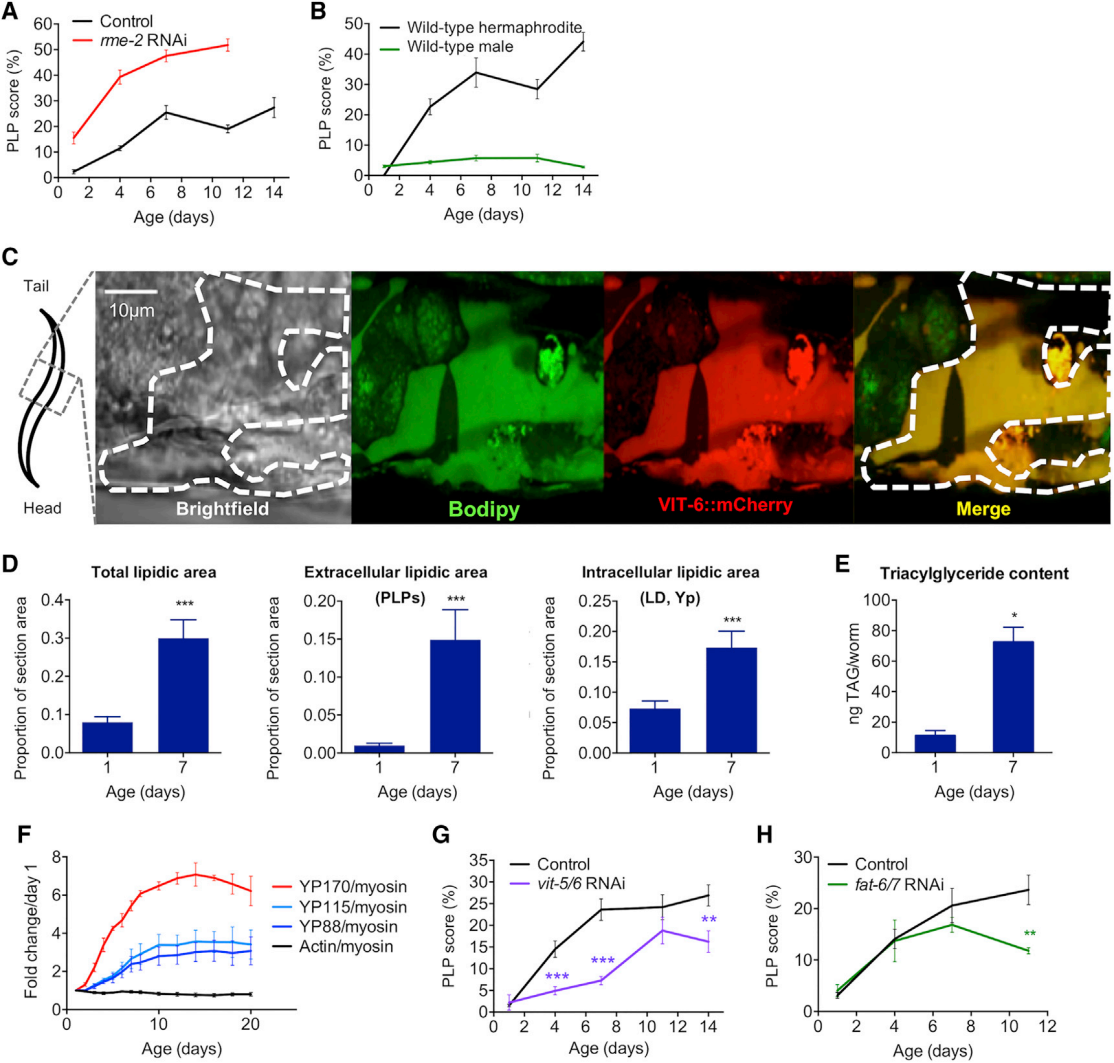
Pathophysiology of Yolk Steatosis in Aging *C. elegans* (A) Inhibition of yolk uptake by *rme-2* RNAi aggravates PLP accumulation; N = 2 trials. (B) Absence of yolk synthesis prevents PLP accumulation in aging males; N = 2 trials. (C) PLPs contain neutral lipids and yolk proteins YP115/YP88 as revealed by co-localization (far right) of Bodipy (center-left) and VIT-6::mCherry (center-right). (D) Estimation of lipid content from measuring intracellular yolk particle (Yp), lipid droplet (LD), and extracellular PLP areas in transmission electron micrographs (TEMs) of mid-body sections reveals a 3.75-fold increase between day 1 and 7 (left), mostly explained by a 15-fold rise in combined PLP area (center) but also by a 3-fold increase in intracellular lipids (right). (E) 8-fold increase in TAG content between day 1 and 7 of adulthood. (F) Lifetime increase in vitellogenins, from SDS-PAGE analysis of wild-type worm lysates. (G) Inhibition of vitellogenin synthesis reduces PLP accumulation. (H) Inhibition of lipid synthesis by *fat-6/7* RNAi reduces PLP accumulation. Worms were raised on *E. coli* B OP50, except for (A), (G), and (H), where *E. coli* K12 HT115 was used. ^∗^p < 0.05, ^∗∗^p < 0.01, ^∗∗∗^p < 0.001. See also Figures S2 and S3 and Data S1 and S2.

PLPs have been previously identified as yolk, as they contain the yolk protein (vitellogenin) VIT-2/YP170 [13, 14]. We verified this by co-localization within the pools of both VIT-2 and VIT-6 (the sole source of YP115 and YP88 in worms; Figure S2C). Consistent with PLPs being yolk, preventing oocyte yolk uptake by RNAi against the receptor *rme-2* accelerated pool growth, whereas males (which do not make yolk) did not accumulate PLPs (Figures 2A and 2B). The magnitude of the pools, which can grow to fill the entire body cavity (Figure 1D, right), and the lipid content of yolk imply a major senescent buildup of lipids in *C. elegans*. Fluorescent labeling of neutral lipids using gentle-fix Bodipy staining [18] confirmed the presence of lipids in PLPs, which co-localized with VIT-6::mCherry (Figures 2C and S2D). Moreover, transmission electron microscopy (TEM) of mid-body sections defined a 3.8-fold increase in lipid organelle area between day 1 and 7 of adulthood (Figure 2D, left), largely attributable to a 15-fold increase in pool area (Figure 2D, center) but also to a 3-fold increase in intracellular lipids (Figure 2D, right). During the same period, a striking 8-fold increase in triacylglyceride (TAG) content was detected, using biochemical assays and lipidomic analysis (Figures 2E, S3A, and S3B; Data S1 and S2). Thus, aging *C. elegans*, by accumulating large amounts of ectopic fat within the pools, become steatotic [16].

### Run-On of Yolk Synthesis Promotes PLP Generation

PLP accumulation has been suggested to result from run-on of lipoprotein production after cessation of egg laying (continued source but loss of sink) [14]. To characterize this putative vitellogenic open faucet, we monitored yolk protein (YP) levels throughout life (days 1–20). This revealed a sustained increase in YP content until day 14, and a maximal 7-fold increase in YP170 (Figures 2F and S4A). Blocking YP accumulation by *vit-5/6* double RNAi (Figures S4A) or inhibiting lipid synthesis by RNAi against the fatty acid desaturases *fat-6* and *fat-7* reduced pool accumulation (Figures 2G and 2H), confirming that PLPs stem from run-on of lipoprotein synthesis.

### Conversion of Intestinal Biomass into Yolk Causes Organ Atrophy

**Figure 3:**
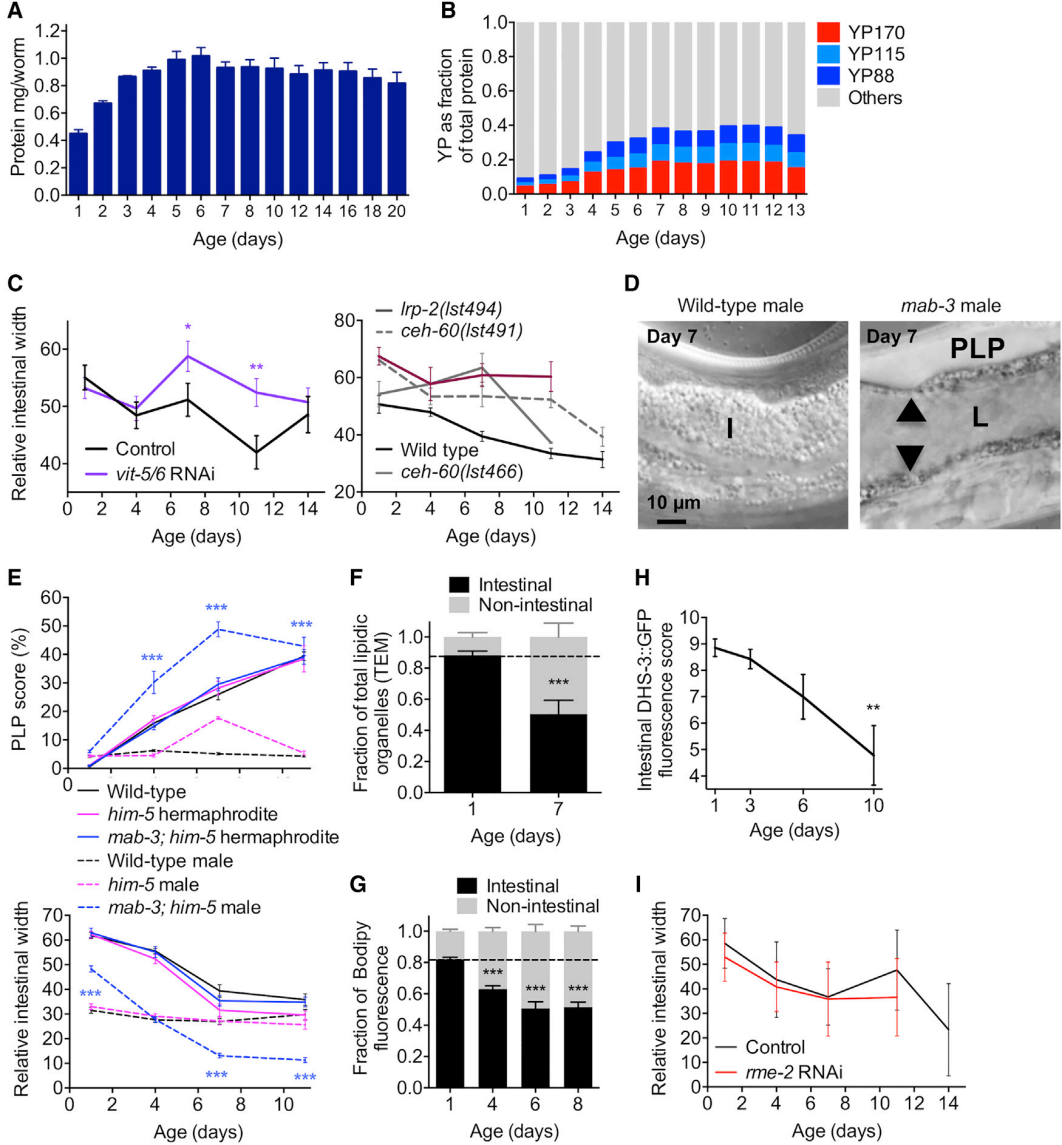
Evidence of Conversion of Intestinal Biomass into Yolk (A) Total protein content of wild-type worms across the course of a lifetime, peaking at day 6. (B) The proportion of YP to total proteins increases 4-fold between day 1 and 7 (gel densitometric analysis). (C) Inhibition of yolk synthesis by *vit-5/6* RNAi (left) or *lrp-2* or *ceh-60* mutations (right) rescues intestinal atrophy; N = 2 trials. All mutants are significantly different from wild-type (p < 0.05) at 2 or more time points. (D) Wild-type males show no gut atrophy (left), whereas yolk-producing *mab-3(mu15)* males exhibit PLPs and gut atrophy (right). (E) Quantitation of PLP accumulation (top) and gut atrophy (bottom) in *mab-3* males; N = 2 trials. Asterisks indicate statistical comparison between *mab-3; him-5* males and *him-5* or wild-type males. (F) TEM of mid-body sections reveals a redistribution of intestinal lipids to other tissues between day 1 and 7. (G) Lipid redistribution confirmed by analysis of neutral lipid staining by Bodipy. (H) Numbers of DHS-3::GFP-marked lipid droplets decrease with age. (I) Inhibition of yolk uptake by *rme-2* RNAi, which aggravates PLP accumulation, does not affect intestinal atrophy. Trials were conducted at 20°C, no FUDR (A–E) or 25°C with FUDR (F–I). ^∗^p < 0.05, ^∗∗^p < 0.01, ^∗∗∗^p < 0.001. See also Figure S4.

Whereas cessation of egg laying after sperm depletion is a pre-requisite for YP accumulation [19], post-reproductive accumulation must reflect continued vitellogenin synthesis. It is notable that *C. elegans* is able to sustain heavy production of lipoprotein to advanced ages, despite the decline with age in feeding rate [20] (Figure S4B). We also noted that although *C. elegans* total protein content plateaus at day 5, the amount of vitellogenin as a proportion of overall protein continues to increase for several more days; strikingly, vitellogenin eventually forms 30%–40% of total worm protein content (Figures 3A, 3B, and S4C). These observations, taken together with the coupling between intestinal atrophy and pool accumulation (Figures 1E and 1F) in all growth conditions tested (altered temperature or bacterial diet, presence of 5-fluoro-2-deoxyuridine (FUDR; Figure S2A), and the fact that the intestine is the site of yolk production [21] suggest a new hypothesis: that the intestine consumes its own biomass to enhance capacity for yolk production.

Consistent with this hypothesis, inhibition of YP production by *vit-5/6* RNAi or by mutation of upstream activators of yolk synthesis (*ceh-60* and *lrp-2*) [22] alleviated both pool accumulation and intestinal atrophy (Figures 2G, 3C, and S4D). Moreover, in wild-type males, which do not make yolk, neither PLP accumulation nor intestinal atrophy was observed. However, induction of ectopic YP production in males by mutation of *mab-3* was sufficient to induce both pathologies (Figures 3D and 3E). Corroborating the idea that intestinal mass is converted into lipoprotein, analysis of lipid distribution by TEM and Bodipy staining [18] revealed that the proportion of intestinal lipids versus PLP lipids decreases between day 1 and 7 (Figures 3F, 3G, and S4E), whereas labeling of intestinal lipid organelles with GFP-tagged DHS-3 (dehydrogenases, short chain-3) [23] becomes patchy and reduced (Figure 3H). Consistent with this, inhibition of YP production by *vit-2* RNAi increases intestinal lipid content [24].

We also considered two alternative scenarios. First, steatosis rather than yolk synthesis might indirectly cause intestinal atrophy. Arguing against this, *rme-2* RNAi, which increases steatosis by inhibiting yolk uptake by oocytes, did not aggravate intestinal atrophy (Figure 3I). Second, the intestinal lumen of older hermaphrodites often appears packed with *E. coli*, raising the possibility that growth of lumenal bacteria promotes gut distension [13]. However, inhibition of *E. coli* proliferation by antibiotics or UV irradiation did not lessen the magnitude of intestinal atrophy and lumenal expansion, although they were delayed (Figures S4F and S4G), ruling out bacterial growth as the main driver of intestinal atrophy.

Altogether, these results strongly suggest that a single etiology, intestinal biomass conversion into lipoprotein, causes three comorbidities in senescing *C. elegans*: intestinal atrophy, extracellular yolk accumulation, and steatotic lipid redistribution.

### Autophagy Promotes Intestinal Atrophy and PLP Accumulation by Gut Biomass Conversion

**Figure 4:**
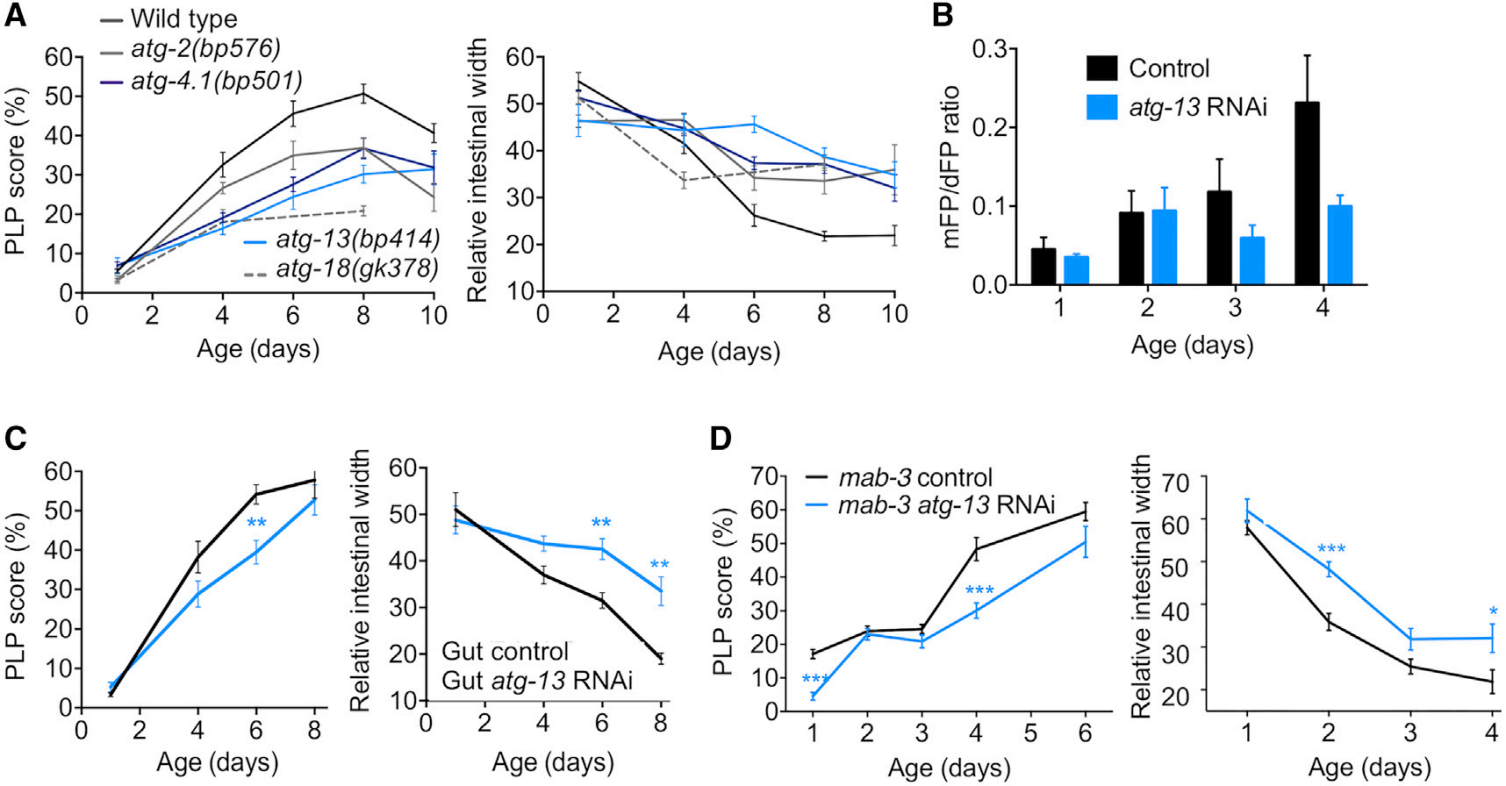
Evidence that Intestinal Autophagy Promotes Intestinal Atrophy and PLP Accumulation (A) Mutations in autophagy genes reduce age-associated PLP accumulation (left) and limit intestinal atrophy (right); N = 2 trials. All mutants were significantly different (p < 0.05) at 2 or more time points. (B) *atg-13* RNAi suppresses the age increase in intestinal autophagy (p = 0.009, 2-way ANOVA). mFP/dFP, monomeric fluorescent protein, dual fluorescent protein. (C) Adult-limited *atg-13* RNAi targeted to the gut reduces age-associated PLP accumulation (left) and intestinal atrophy (right); N = 2 trials. (D) Adult-limited *atg-13* RNAi rescues PLP accumulation and gut atrophy in *mab-3* males; N = 3 trials. ^∗^p < 0.05, ^∗∗^p < 0.01, ^∗∗∗^p < 0.001. See also Figure S5.

But how does this biomass conversion occur? Such a process would require bulk breakdown and recycling of cellular components, a role typically fulfilled by autophagy (macroautophagy, specifically) in contexts of starvation, ecdysozoan molting, and metamorphosis [25]. Notably, levels of autophagy appear to be high in the intestine of adult hermaphrodites [26]. To test this hypothesis, we first examined mutants defective in induction of autophagy (*atg-13(bp414)*), autophagosomal vesicle elongation (*atg-4.1(bp501)*), and Atg9p retrieval (*atg-2(bp576)* and *atg-18(gk378)*), and found that all four mutations reduced both gut atrophy and pool accumulation (Figure 4A). This implies that autophagy promotes both pathologies.

To explore how autophagy inhibition suppresses gut atrophy, we tested effects of adult-specific inhibition of autophagy by *atg-13* RNAi, selecting *atg-13* [27] because, as an upstream regulator of autophagy, its inhibition may be less likely to induce deleterious pleiotropic effects through accumulation of abnormal autophagosomes. Using an intestine-specific reporter of autolysosome formation [26], we first obtained evidence that adult-specific *atg-13* RNAi reduces intestinal autophagy (Figure 4B). We next found that adult-specific *atg-13* RNAi reduced both yolk pool accumulation and intestinal atrophy when broadly applied (Figure S5A), but also when restricted to the adult intestine (Figure 4C). Moreover, adult-limited *atg-13* RNAi rescued pathologies induced by intestinal feminization in *mab-3* males (Figure 4D). Conversely, gut-specific transgenic rescue of the *atg-13(bp414)* mutation restored intestinal atrophy without a detectable increase in PLP accumulation (Figure S5B).

**Figure 5:**
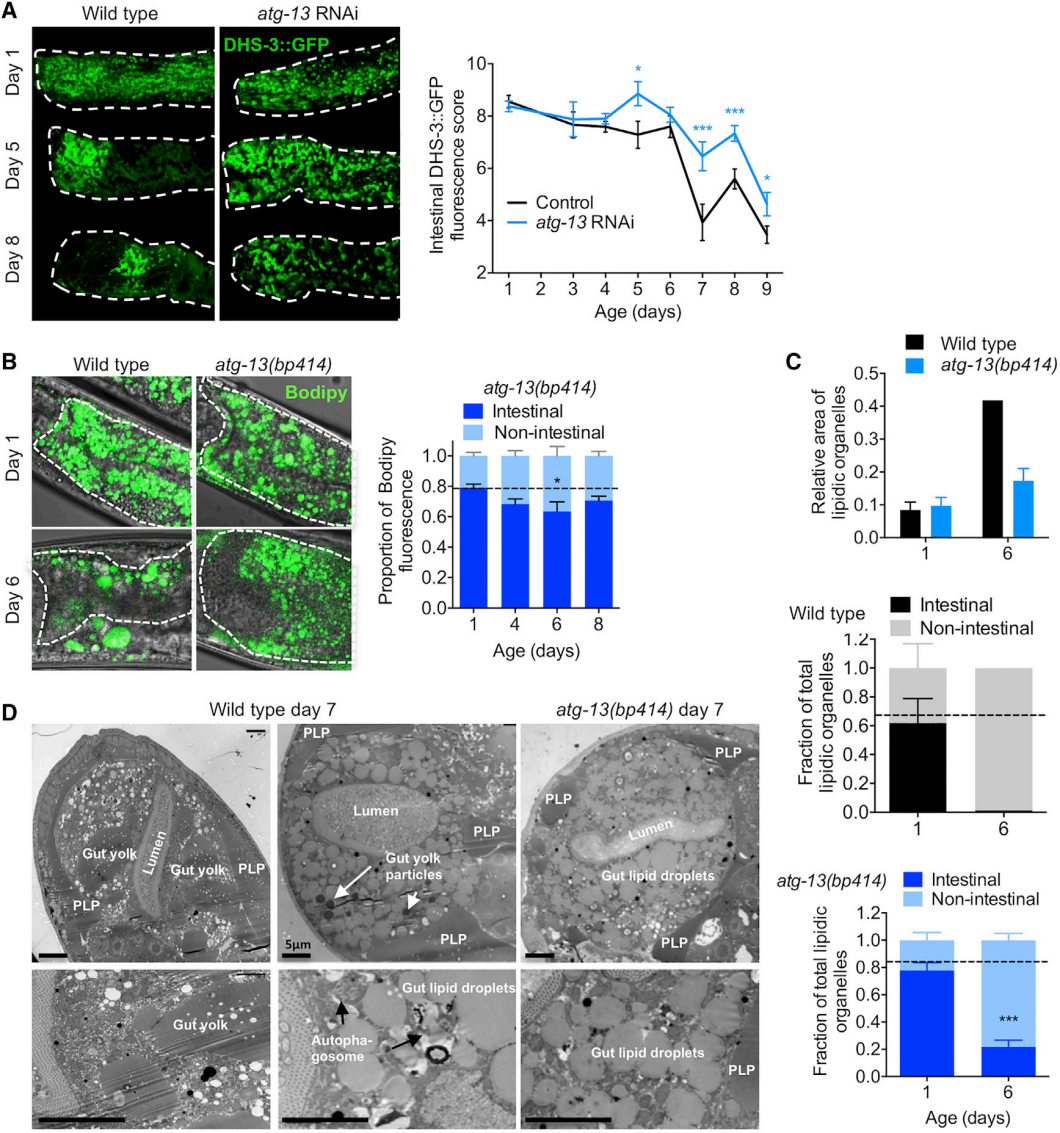
Inhibition of *atg-13* Reduces Age-Associated Lipid Redistribution (A) Adult-limited *atg-13* RNAi delays the age-associated decrease in DHS-3::GFP-labeled intestinal lipid droplets. Images showing labeling of intestinal lipid droplets using DHS-3::GFP in control and *atg-13* RNAi animals (left). Quantification of DHS-3::GFP-labeled intestinal lipid droplets (right). (B) Age-associated lipid redistribution is inhibited by *atg-13(bp414)*. Bodipy staining of neutral lipids in control and *atg-13* RNAi animals reveals preservation of Bodipy-labeled intestinal lipid content (cf. Figure 3G). (C) Quantification of lipidic organelles using TEM. Age-related increase in lipidic area is suppressed by *atg-13(bp414)* (top). Relative intestinal lipidic organelle content is preserved during aging in *atg-13(bp414)* compared to wild-type (middle and bottom). (D) TEM images at 3,000× (top) and 10,000× (bottom) of wild-type (left and center) and *atg-13(bp414)* (right) worm sections on day 7 of adulthood. *atg-13(bp414)* worms have reduced PLPs and increased intestinal lipid droplets compared to wild-type. ^∗^p < 0.05, ^∗∗∗^p < 0.001. See also Table S1.

Abrogation of *atg-13*, *atg-4.1*, *atg-9*, *atg-2*, or *atg-18* by means of mutation or RNAi also impeded senescent lipid redistribution, as shown by DHS-3::GFP [23], Bodipy [18], and TEM lipid organelle analyses (Figures 5, S5C, and S5D). Adult-specific RNAi of *atg-2*, *atg-4.1*, *atg-9*, and *atg-18*, like that of *atg-13*, also reduced gut atrophy (Figure S5E). Moreover, *atg-13(bp414)* largely suppressed age changes in lipid profiles (Figure S5F; Table S1). As autophagosomes consume endomembranes, we followed gut intracellular markers for Golgi and early, late, and recycling endosomes during early aging. We failed to see salient age-related changes in endosomal staining, but we did observe a significant reduction in intestinal Golgi labeling by alpha-mannosidase II-GFP at day 10 [28], which was rescued by *atg-13* RNAi (Figure S5G). This is consistent with the hypothesis that autophagosomes compete with the Golgi apparatus for endomembrane availability [29]. These results suggest that *atg-13*-mediated lipophagy promotes the conversion of intestinal lipids into yolk lipids destined for export. Taken together, our results suggest that autophagy promotes gut-to-yolk biomass conversion, leading to senescent multimorbidity.

### Gut Biomass Conversion Involves a Trade-Off between Fitness and Late-Life Health

**Figure 6:**
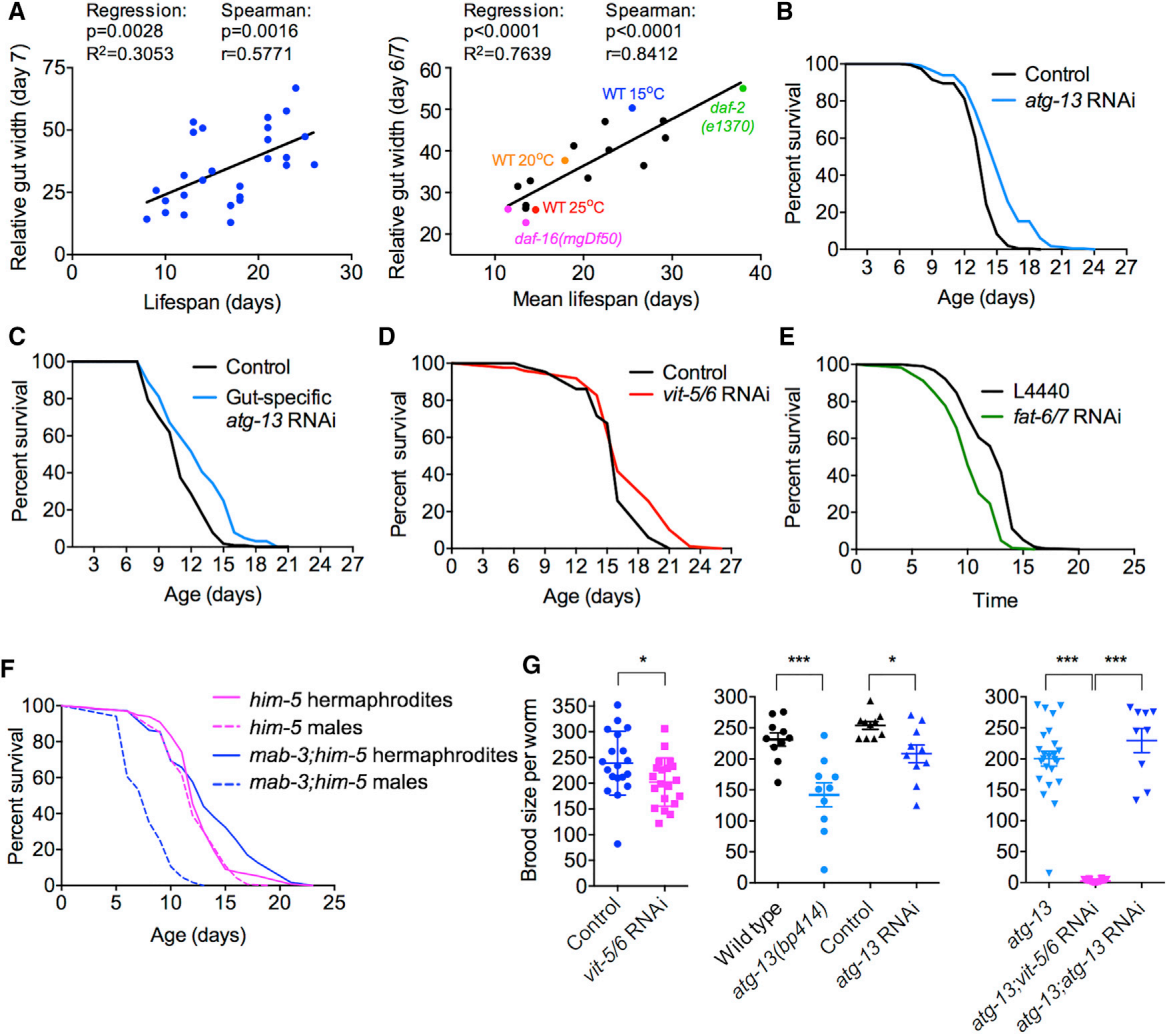
Intestinal Autophagy Promotes Optimal Reproduction at the Expense of Longevity (A) Correlation between intestinal atrophy and lifespan. Left: individual worm analysis, day 7 gut width. Right: worm population analysis, different treatments affecting lifespan, day 6/7 gut width. (B) Adult-limited *atg-13* RNAi targeted to the whole animal extends lifespan; N = 6 trials. (C) Adult- and gut-limited *atg-13* RNAi extends lifespan; N = 5 trials. (D) Inhibition of yolk synthesis extends lifespan; N = 5 trials. (E) Inhibition of fatty acid synthesis shortens lifespan; N = 2 trials. Previously, no effect of *fat-6(tm331); fat-7(wa36)* on lifespan was detected [51]; the reason for the different effects of *fat-6/7* RNAi and mutation remains unknown. (F) *mab-3; him-5* males are short lived but *mab-3; him-5* hermaphrodites are not; N = 2 trials. Reduced lifespan of *mab-3* males could be attributable to the visceral or the tail abnormalities. (G) Adult-limited *vit-5/6* RNAi (left) or *atg-13* RNAi and *atg-13* mutation (center) each modestly reduces brood size, but combined *vit-5/6* RNAi and *atg-13(bp414)* cause sterility (right); N = 2 trials. Experiments were performed at 20°C without FUDR (A and F), 25°C without FUDR (D, E, and G), and 25°C with FUDR (B and C). ^∗^p < 0.05, ^∗∗∗^p < 0.001. See also Figure S6 and Data S3.

We then explored whether gut-to-yolk biomass conversion can contribute to late-life mortality. Analysis of age-specific pathology measures and survival in individual worms (Figure 6A, left) and in various strains and growth conditions (Figure 6A, right) identified a strong positive correlation between lifespan and intestinal width in mid-life, suggesting that intestinal atrophy can contribute to mortality. Inhibiting gut-to-yolk biomass conversion by *atg-13* RNAi targeted to the whole adult animal or to the intestine alone extended lifespan at 25°C (+21.3% and +5.4%, respectively, p < 0.0001 in each case, log rank test; Figures 6B and 6C), but not at 20°C (Data S3). At 25°C, lifespan was also increased by adult-limited, whole-worm RNAi of *atg-2* (+9.7%, p < 0.0001) but not *atg-18* (Figure S5H). These results could reflect condition dependency with respect to which senescent pathologies are life limiting (Figure 1A). *vit-5/6* RNAi also significantly increased lifespan (+12.8%, p < 0.0001; Figure 6D; Data S3), as reported for *vit-5* RNAi [30], whereas *rme-2* RNAi, which increases PLP accumulation (Figure 2A), reduced lifespan (−11.6%, p < 0.0001; Figure S5I; Data S3). *fat-6/7* RNAi caused marked acceleration in gut atrophy rate (Figure S5J), which may reflect increased biomass conversion in response to reduced *de novo* fatty acid synthesis, and also reduced lifespan (−14.0%, p < 0.0001; Figure 6E). Similarly, *mab-3(mu15)* males, which have severe intestinal atrophy, were short lived (−39.6%, p < 0.0001), whereas *mab-3* hermaphrodites were not (Figure 6F; Data S3). Taken together, these results support the view that intestinal atrophy and PLP accumulation can contribute to late-life mortality.

Although *vit-5/6* RNAi, *atg-13* RNAi, and *atg-13(bp414)* had little effect on brood size, consistent with the normal fertility of *ceh-60* and *lrp-2* mutants [22], *vit-5/6* RNAi in *atg-13(bp414)* worms caused near-sterility (Figure 6G). These results suggest that high levels of autophagy in the intestine promote fitness by supporting yolk production in early adulthood (during reproduction), whereas run-on of intestinal autophagy contributes to gut atrophy and pool formation in later life (post-reproduction). This is further supported by the fact that mated hermaphrodites with increased brood sizes display accelerated gut atrophy (Figure S6A) consistent with life-shortening effects of exposure to males [31] but exhibit delayed PLP accumulation, most likely due to prolonged egg laying (increased yolk sink; Figure S6B). Exposure to male secretions alone did not affect either visceral pathology (Figures S6C–S6H), consistent with recent evidence that exposure to males shortens lifespan by two distinct mechanisms [31]. All this supports the existence of a trade-off between beneficial effects of biomass conversion on reproductive fitness and later-life pathogenic effects.

### Insulin/IGF-1 Signaling (IIS) Promotes Gut-to-Yolk Biomass Conversion

**Figure 7:**
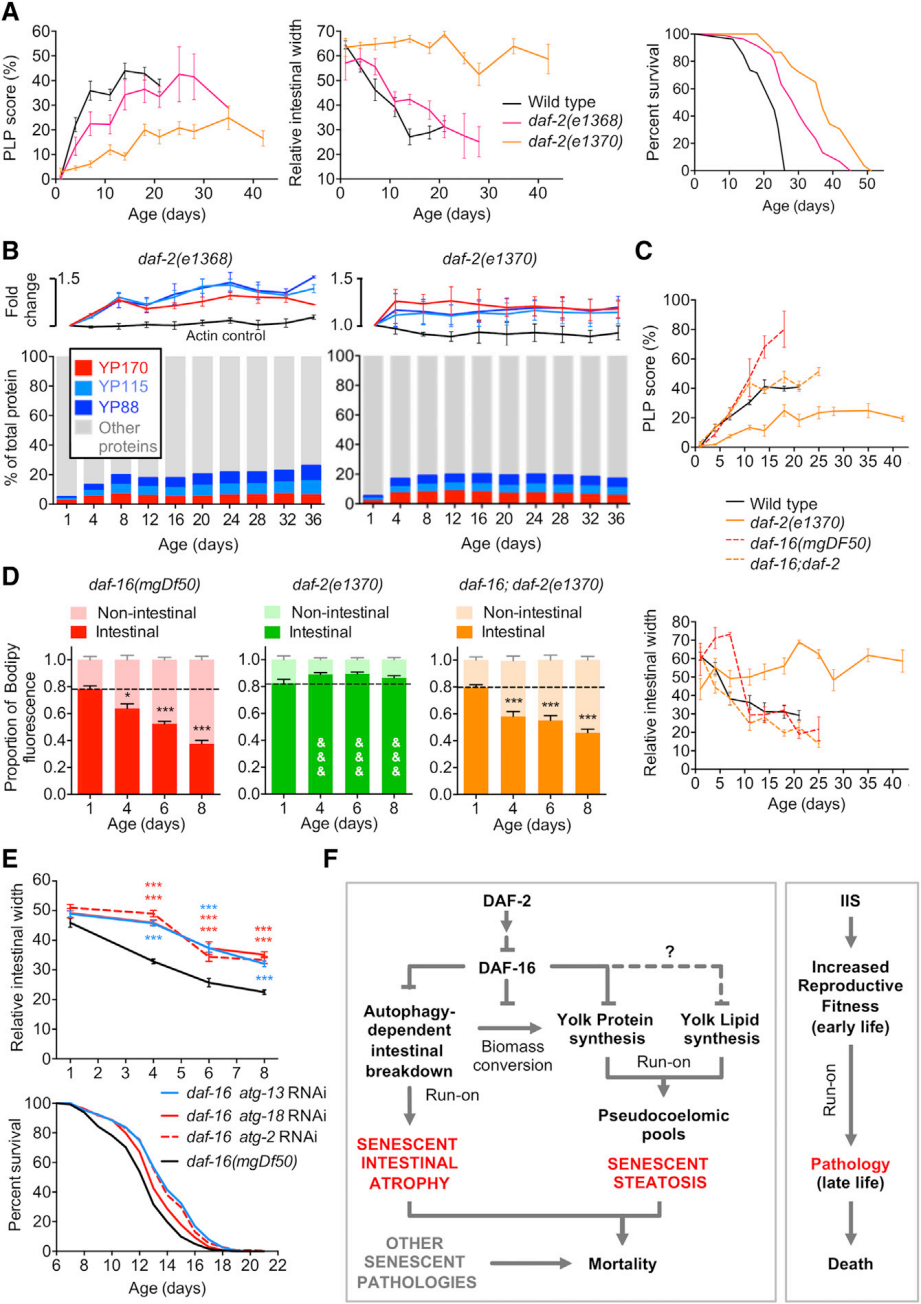
Reduced Insulin/IGF-1 Signaling Inhibits Gut-to-Yolk Biomass Conversion (A) PLP accumulation (left) and intestinal atrophy (center) are suppressed modestly by *daf-2(e1368)* and strongly by *daf-2(e1370)*, which also has a larger effect on lifespan (right), confirming findings in a recent study [52]. (B) YP levels and YP as proportion of total protein in *daf-2(e1368)* (left) and *daf-2(e1370)* (right) (cf. Figures 2F and 3B). (C) PLP accumulation (top) and intestinal atrophy (bottom) in *daf-2(e1370)* require *daf-16*; N = 2 trials. (D) Age-associated neutral lipid redistribution (Bodipy) is suppressed in *daf-2(e1370)* mutants, and this requires *daf-16*. ^∗^p < 0.05, ^∗∗∗^p < 0.001, ^&&&^p < 0.001, compared to age-matched *daf-16*, *daf-16; daf-2*, or wild-type (cf. Figure 3G). (E) RNAi of *atg-2*, *atg-13*, and *atg-18* all suppress intestinal atrophy (top), whereas *atg-2* and *atg-13* also increase lifespan (bottom; p < 0.01 all trials; N = 2 and 5, respectively) in a *daf-16(mgDf50)* mutant (25°C, FUDR). ^∗∗∗^p < 0.001. (F) Proposed mechanism of antagonistically pleiotropic action of IIS promoting reproduction at the expense of senescent pathologies, through gut-to-yolk biomass conversion. See also Figure S7.

Another factor that enhances evolutionary fitness at the expense of senescent pathologies and lifespan shortening is IIS, which promotes reproductive development [3, 13]. Hence, the wild-type *daf-2* insulin/IGF-1 receptor allele is a good example of antagonistic pleiotropy (AP) [10]. The proximate mechanisms downstream of IIS that account for effects on lifespan have been sought for many years, e.g., by detailed characterization of IIS-regulated genes, yet remain uncertain. One possibility is that gut-to-yolk biomass conversion is one such proximate mechanism. Consistent with this idea, mutation of *daf-2* not only extends lifespan [3] but also rescues senescent multimorbidity, including YP increase, PLP accumulation, intestinal atrophy, and lipid redistribution (Figures 7A–7D and S7A–S7C) [13, 19], which requires the DAF-16/FoxO transcription factor (Figures 7C, 7D, and S7C) [3, 19].

Another intervention that extends lifespan in a *daf-16*-dependent manner is removal of the germline, which also triggers DAF-16 nuclear localization most prominently in intestinal cells [3]. Blocking germline development using *glp-4(bn2ts)* also delayed intestinal atrophy, and this effect was largely *daf-16* dependent (Figure S7D), suggesting that DAF-16 suppresses gut-to-yolk biomass conversion in this context also. Moreover, in *daf-16(mgDf50)* mutants, *atg-2*, *atg-13*, and *atg-18* RNAi rescued intestinal atrophy to a similar extent as in wild-type worms (Figure 7E, top), whereas *atg-2* and *atg-13* RNAi extended mean lifespan (+11.7% and +12.6%, respectively, both p < 0.0001; Figures 7E and S7E; Data S3), suggesting that autophagy acts in parallel to or downstream of DAF-16. Full understanding of how IIS regulates gut-to-yolk biomass conversion will require further study.

## Discussion

These findings support a model in which the run-on of reproductive functions promotes senescent pathogenesis in *C. elegans* hermaphrodites. This involves IIS-driven and autophagy-mediated conversion of intestinal biomass into yolk and, subsequently, steatotic yolk pools (PLPs). The model proposes a single etiology for several major comorbidities of worm aging, and identifies a mechanism by which IIS, through inhibition of DAF-16, can cause senescent atrophy of the hermaphrodite intestine (Figure 7F).

The mechanisms proposed here differ from the traditional view of aging as caused by homeostatic imbalance and damage accumulation. Here instead, persistence of function specified by wild-type genes beyond their “intended” purpose actively drives senescent pathogenesis, consistent with the hyper-function model of AP action [5, 8] rather than the disposable soma model [32].

### Coordinated Development of Senescent Pathologies in Mid-Adulthood

A survey of patterns of development of major senescent pathologies revealed that these appear not, as expected, near the end of life, but in mid-adulthood. The overall pattern suggests a mid-life phase characterized by vigorous development of pathologies, ending at around d10–d12 of adulthood (Figures 1B and 1C). The major anatomical changes that result most likely contribute to reported age changes in *C. elegans* expression profiles, e.g., levels of intestinal proteins decline with age [33]. Thus, many age changes in mRNA and protein level may be the consequence rather than the cause of pathogenic processes such as gut-to-yolk biomass conversion. One possibility is that when the major pathologies have reached their developmental endpoints around d10–d12 the nematodes are, as a consequence, terminally ill and spend the rest of their lives slowly dying.

### PLPs as a Form of Senescent Obesity in *C. elegans*

Yolk accumulation in adult *C. elegans* has been described previously, but not its full extent during aging. Here we report that age increases in vitellogenin levels reach 7-fold that of young adults, forming up to 30%–40% of total worm protein, whereas TAG levels increase ∼8-fold. The presence of large yolky pools staining with the lipid dye Bodipy implies that such pools are major extracellular lipid deposits, helping to account for the age increase in TAG. The severely steatotic nature of elderly *C. elegans* described here and elsewhere [16] could have been overlooked previously due to the use in lipid staining protocols of fixation methods with dehydration steps; here we used a gentle staining method without such steps [18].

We postulate that PLPs develop by run-on of yolk lipid production (Figure 7F), but cannot exclude a contribution from lipids released by degeneration of other organs such as the distal gonad [9]. The age-dependent accumulation and gross appearance of the PLPs suggest that they represent a form of pathological senescent obesity in *C. elegans*. Consistent with this, lifespan was slightly reduced by knockdown of expression of the yolk receptor *rme-2*, which accelerated PLP accumulation without affecting intestinal atrophy (Figures 2A, 3I, and S5I). A possible explanation for PLP toxicity is late-life ectopic deposition, e.g., in muscle and epidermis (data not shown) [14], due to PLP endocytosis as part of a senescent redistribution of fat stores. Such age-associated redistribution of fat is reminiscent of pathogenic shifts in fat distribution that occur in aging mammals, underscoring the potential of *C. elegans* as a model for investigating pathologies of fat metabolism.

### Gut-to-Yolk Biomass Conversion Causes Intestinal Senescence

In mammals, senescence causes diverse pathologies. Important questions are: to what extent do multiple senescent pathologies originate from common etiologies [12]? And: what is the nature of these etiologies? Here we provide an example of how one etiology, gut-to-yolk biomass conversion, promotes two major senescent pathologies: intestinal atrophy and yolk accumulation, each of which may lead to further pathologies. For example, yolk accumulation promotes uterine tumor development [34].

The intestine is the major somatic internal organ in *C. elegans*, also serving as liver and adipose tissue. As *C. elegans* hermaphrodites get old, the intestine undergoes severe deterioration [15], most likely impairing worm viability. Consistent with this, the extent of intestinal atrophy in early to mid-adulthood is predictive of lifespan (Figure 6A). Furthermore, the lifespan-controlling transcription factor DAF-16 exerts its effects in the intestine [17]. Moreover, intestinal necrosis is a key event in organismal death [35]. All this is in agreement with the view that intestinal senescent pathology can be life limiting to *C. elegans*. Consistent with this, we document here that lifespan can be increased by treatments that reduce intestinal atrophy (e.g., inhibition of genes promoting vitellogenesis and autophagy) and reduced by treatments that increase intestinal atrophy (blocking *de novo* fat synthesis and induction of yolk synthesis in the male gut).

### Autophagy as a Promoter of Gut-to-Yolk Biomass Conversion and Intestinal Pathology

Our findings suggest that autophagy promotes intestinal atrophy and yolk steatosis, consistent with gut-to-yolk biomass conversion. Yet this result was unexpected, insofar as the role of autophagy in aging is often viewed as a protective one, congruent with the idea that aging results from molecular damage: by removing damaged cellular components, autophagy helps to maintain the cell in a youthful state [36]. Consistent with this, inhibition of genes encoding autophagy-related proteins can reduce extended longevity in *C. elegans*, e.g., when induced by mutation of *daf-2*, dietary restriction, and germline loss [36]. Moreover, it has been proposed that autophagy is increased in *daf-2* mutants, contributing to their increased longevity [36, 37]. Yet our findings imply that gut-to-yolk biomass conversion is reduced in *daf-2* mutants, suggesting reduced autophagy. How may such different claims be reconciled?

The idea that autophagy might promote rather than suppress senescent changes is not in itself contentious. Autophagy can enhance as well as inhibit the development of pathologies, including senescent ones [38]. For example, production of the senescence-associated secretory phenotype (SASP) proteins in mammalian cells is promoted by autophagy [39], there playing a role somewhat similar to that in *C. elegans* yolk production proposed here.

Inhibition of genes involved in autophagy has been observed either to reduce or increase *C. elegans* lifespan, or to have no effect on it [36, 40, 41] (this study). We postulate that the pathologies that limit life vary with culture conditions and genotype (Figure 1A), and between species. Thus, the effect on lifespan of inhibiting autophagy will depend on whether life-limiting pathologies are inhibited or enhanced by autophagy. For example, we found that *atg-13* RNAi consistently increased lifespan at 25°C, but not at 20°C, despite ameliorating senescent pathologies at both temperatures (Figures 4A, 4C, 6B, 6C, and S5A; Data S3). In another study, where RNAi was performed on 14 autophagy genes and in many cases increased lifespan [40], a high concentration of 5-fluoro-2-deoxyuridine (FUDR) was used. Given that intestinal autophagy can protect *C. elegans* against bacterial infection [42], a possibility is that FUDR can alleviate life-limiting infection by co-cultured *E. coli* that autophagy protects against; autophagy also appears to protect *C. elegans* against end-of-life loss of intestinal barrier function [43].

Autophagy may also exert life-shortening and life-extending effects at different times in *C. elegans* life history. Reduced autophagy during development can impair health [40, 44], whereas late-life knockdown of autophagy genes can increase lifespan [41]. Effects of autophagy knockdown on lifespan may also depend upon severity of knockdown. The adult hermaphrodite intestine exhibits relatively high levels of autophagy [26] and protein turnover [45]; we speculate that high intestinal autophagy levels that maximize yolk production exceed homeostatic requirements, such that partial inhibition of autophagy can improve late-life health, whereas full abrogation impairs essential housekeeping functions and reduces health. More generally, where wild-type levels of autophagy promote life-limiting pathology, moderate inhibition in autophagy may extend lifespan in the wild-type but shorten it in a long-lived mutant in which autophagy levels are already reduced [45] to a level that is optimal to prevent pathology.

### Does IIS Increase or Decrease Intestinal Autophagy?

This study suggests a mechanism by which IIS causes visceral pathology: autophagy-dependent gut-to-yolk biomass conversion (Figure 7F, left). This is different from a prior suggestion that inhibition of autophagy by IIS promotes aging. The status of autophagy in *daf-2* mutants remains unclear. Fully reliable means to directly measure autophagic activity in *C. elegans* have yet to be developed [37, 44]. For example, autophagosome numbers in *daf-2* mutants are increased in the larval hypodermis and in the adult intestine [36, 37]. This could reflect either increased autophagosome production (increased autophagy) or a slowing down in autophagosome consumption (decreased autophagy) [37, 44]. However, autophagy does not appear to be blocked in the *daf-2* mutant intestine (at least, not entirely), which could imply increased autophagy [37]. Another broad indicator of autophagic activity is protein turnover rate (although this is also a function of ubiquitin/proteasomal activity). For example, aging *C. elegans* show a decline in both autophagy [37] and protein turnover [45]. However, *daf-2* mutants show major reductions in both protein synthesis [46] and turnover [47, 48]. Thus, different forms of evidence suggest either increased or decreased autophagy in *daf-2* mutants. Our findings support the latter view and suggest a mechanism by which autophagy acts to promote intestinal senescence (gut-to-yolk biomass conversion). Given that IIS strongly promotes yolk production [19], intestinal atrophy could result from the combined effects of IIS-promoted dominance of the translational machinery by vitellogenin synthesis, constitutively high levels of intestinal autophagy [26, 45], and yolk export [21]. Another possibility is that IIS promotes coupled protein synthesis and autophagy [39]. In conclusion, this study provides a new perspective on the role of autophagy in *C. elegans* aging, which we hope will usefully inform future investigations of the topic.

### Gut-to-Yolk Biomass Conversion: A Mechanism for *daf-2* Antagonistic Pleiotropy

Aging evolves at least in part as the result of antagonistic pleiotropy (AP), implying that late-life gene action causes senescence, including senescent pathologies [5, 8]. *daf-2* is a gene with strong AP effects, promoting reproductive growth in early life and senescent pathology in later life [10]. The finding that IIS promotes gut-to-yolk biomass conversion provides a mechanistic account of how IIS-determined AP is enacted, as follows. In early adulthood, IIS promotes gut-to-yolk biomass conversion, which increases reproductive fitness by boosting yolk production capacity. Supporting this, combined knockdown of autophagy and vitellogenesis strongly reduces fertility (Figure 6G), and protein turnover rate is reduced in *daf-2* mutants [46, 47]. Continued gut-to-yolk biomass conversion later in adulthood leads to pathologies in the form of intestinal atrophy and yolk-derived PLPs, which can shorten lifespan (Figure 7F, right).

Our findings raise the question: why do hermaphrodites not turn off gut-to-yolk biomass conversion after cessation of reproduction? Here the evolutionary theory suggests a possible answer: that such an off switch did not evolve due to the reduced force of natural selection in post-reproductive animals. By this view, biomass conversion is simply left on, in an open-faucet-type mechanism, an example of process run-on or hyper-function [5, 9, 14], where continued yolk production after reproduction in self-fertilizing hermaphrodites is futile in fitness terms. However, it remains possible that later yolk production does promote fitness, e.g., in mated hermaphrodites, which have a longer reproductive period.

Run-on also contributes to other *C. elegans* senescent pathologies that are promoted by IIS, including run-on of embryogenetic programs in unfertilized oocytes, which promotes tumor development [34, 49], and germline apoptosis, which promotes gonad atrophy [9]. That IIS promotes mechanistically very distinct hyper-functions as part of the hermaphroditic reproductive program suggests that run-on mechanisms may be typical of the way that IIS promotes aging.

### Conclusions

This study defines a syndrome of senescent multimorbidity driven by wild-type gene action in which atrophy of a source tissue is coupled by ectopic fat deposition to pathology in other, sink tissues. Possible generalization of this type of etiology is suggested by parallels with bone erosion in lactating mammals, which ensures sufficient calcium in milk [50]. In humans, menopause-associated run-on of such bone-to-milk calcium transfer may contribute to both osteoporosis and conditions promoted by ectopic Ca^2+^ deposition, such as vascular calcification and osteoarthritis. More broadly, these investigations demonstrate how senescent pathologies can be generated by the futile sustained activity of wild-type biological programs [5, 8], rather than by passive and stochastic wear and tear processes.

## STAR★Methods

### Key Resources Table

**Table:** 

REAGENT or RESOURCE	SOURCE	IDENTIFIER
**Bacterial and Virus Strains**		

OP50 *Escherichia coli* B strain (Uracil auxotroph)	*Caenorhabditis* Genetics Center	OP50
PY79 *Bacillus subtilis* strain	F. Cabreiro, University College London	PY79

**Critical Commercial Assays**		

Triglyceride Quantification Colorimetric/Fluorometric Kit	BioVision, Milpitas, California	#K622
BODIPY 493/503 (4,4-difluoro-1,3,5,7,8-pentamethyl-4-bora-3a,4a-diaza-s-indacene)	Fisher Scientific, Loughborough, United Kingdom	#D3922
Sample Buffer, Laemmli 2X Concentrate	Sigma, St Louis, Missouri	#S3401
Criterion XT pre-cast gels 4-12% Bis-Tris	Biorad, Hercules, California	#3450124
XT MOPS running buffer	Biorad, Hercules, California	#1610788
CelLytic M	Biorad, Hercules, California	#C2978

**Experimental Models: Organisms/Strains**		

N2 wild type	*Caenorhabditis* Genetics Center	N2
CB3168 *him-1(e879); mab-3(e1240)*	*Caenorhabditis* Genetics Center	CB3168
CF80 *mab-3(mu15); him-5(e1490)*	*Caenorhabditis* Genetics Center	CF80
DH26 *rrf-3(b26)*	*Caenorhabditis* Genetics Center	DH26
DR466 *him-5(e1490)*	*Caenorhabditis* Genetics Center	DR466
DR1563 *daf-2(e1370)*	*Caenorhabditis* Genetics Center	DR1563
DR1572 *daf-2(e1368)*	*Caenorhabditis* Genetics Center	DR1572
GA507 *glp-4(bn2) daf-16(mgDf50)*	D. Gems, University College London	GA507
GA1500 *bIs1[pvit-2::vit-2::GFP + rol-6(su1006)]*	D. Gems, University College London	GA1500
GR1307 *daf-16(mgDf50)*	*Caenorhabditis* Genetics Center	GR1307
GA1711 *wuEx272[pvit-6::vit-6::mCherry + rol-6]*	D. Gems, University College London	GA1711
GA1715 *wuEx280[rol-6]*	D. Gems, University College London	GA1715
GA1729 *atg-13(bp414)*;*wuEx292[pges-1::atg-13 + rol-6]*	D. Gems, University College London	GA1729
GA1730 *atg-13(bp414); wuEx293[pges-1::atg-13 + rol-6]*	D. Gems, University College London	GA1730
GA2100 *bIs1[pvit-2::vit-2::GFP + rol-6]; wuEx277[pvit-6::vit-6::mCherry + rol-6*]	D. Gems, University College London	GA2100
HZ1683 *him-5(e1490); atg-2(bp576)*	*Caenorhabditis* Genetics Center	HZ1683
HZ1685 *atg-4.1(bp501)*	*Caenorhabditis* Genetics Center	HZ1685
HZ1688 *atg-13(bp414)*	*Caenorhabditis* Genetics Center	HZ1688
LIU1 *ldrIs1 [pdhs-3::dhs-3::GFP + unc-76(+)*]	*Caenorhabditis* Genetics Center	LIU1
LSC897 *ceh-60(lst466)*	L. Temmerman, KU Leuven	LSC897
LSC903 *ceh-60(lst491)*	L. Temmerman, KU Leuven	LSC903
LSC904 *lrp-2(lst464)*	L. Temmerman, KU Leuven	LSC904
RT311 *unc-119(ed3); pwIs69 [vha6p::GFP::rab-11 + unc-119(+)]*	*Caenorhabditis* Genetics Center	RT311
RT476 *unc-119(ed3); pwIs170 [vha6p::GFP::rab-7 + Cbr-unc-119(+)]*	*Caenorhabditis* Genetics Center	RT476
RT525 *unc-119(ed3); pwIs206 [vha6p::GFP::rab-10 + Cbr-unc-119(+)]*	*Caenorhabditis* Genetics Center	RT525
RT1315 *unc-119(ed3); pwIs503 [pvha-6::mans::GFP + Cbr-unc-119(+)]*	*Caenorhabditis* Genetics Center	RT1315
SS104 *glp-4(bn2)*	*Caenorhabditis* Genetics Center	SS104
VC893 *atg-18(gk378)*	*Caenorhabditis* Genetics Center	VC893
VP303 *rde-1(ne219); kbEx200 [pnhx-2::rde-1]*	*Caenorhabditis* Genetics Center	VP303

### Contact for Reagent and Resource Sharing

Further information and requests for resources and reagents should be directed to and will be fulfilled by the Lead Contact, David Gems (david.gems@ucl.ac.uk).

### Experimental Models and Subject Details

#### *C. elegans* Culture and Strains

*C. elegans* were maintained under standard conditions [53], at 20°C on NGM plates seeded with *Escherichia coli* OP50, unless otherwise stated. For *Bacillus subtilis* trials the PY79 strain was used. Some RNAi experiments were performed at 25°C. Hermaphrodites were used except when otherwise stated. Males were cultured at low density (∼5 per plate) during aging trials to reduce detrimental effects of male-male interactions [54].

The following strains were used: N2 (wild-type N2 male stock, N2 CGCM) [55]; CB3168 *him-1(e879); mab-3(e1240)* [56], CF80 *mab-3(mu15); him-5(e1490)*, DH26 *rrf-3(b26)*, DR466 *him-5(e1490)*, DR1563 *daf-2(e1370)*, DR1572 *daf-2(e1368)*, GA507 *glp-4(bn2) daf-16(mgDf50),* GA1500 *bIs1[pvit-2::vit-2::GFP + rol-6(su1006)]*, GR1307 *daf-16(mgDf50)*, HZ1683 *him-5(e1490); atg-2(bp576)*, HZ1685 *atg-4.1(bp501)*, HZ1688 *atg-13(bp414)*, LIU1 *ldrIs1 [pdhs-3::dhs-3::GFP + unc-76(+)*], LSC897 *ceh-60(lst466)*, LSC903 *ceh-60(lst491)*, LSC904 *lrp-2(lst464)*, RT311 *unc-119(ed3); pwIs69 [vha6p::GFP::rab-11 + unc-119(+)]*, RT476 *unc-119(ed3); pwIs170 [vha6p::GFP::rab-7 + Cbr-unc-119(+)]*, RT525 *unc-119(ed3); pwIs206 [vha6p::GFP::rab-10 + Cbr-unc-119(+)],* RT1315 *unc-119(ed3); pwIs503 [pvha-6::mans::GFP + Cbr-unc-119(+)]*, SS104 *glp-4(bn2)*, VC893 *atg-18(gk378)*, VP303 *rde-1(ne219); kbEx200 [pnhx-2::rde-1]*. The following strains were created for this study: GA1711 *wuEx272 [pvit-6::vit-6::mCherry + rol-6]*, GA1715 *wuEx280[rol-6]*, GA1729 *atg-13(bp414)*; *wuEx292[pges-1::atg-13 + rol-6]*, GA1730 *atg-13(bp414); wuEx293[pges-1::atg-13 + rol-6],* GA2100 *bIs1[pvit-2::vit-2::GFP + rol-6]; wuEx277[pvit-6::vit-6::mCherry + rol-6*].

### Method Details

#### Pathology Measurements

Worms were mounted onto 2% agar pads and anesthetized with 0.2% levamisole. Nomarski microscopy images were acquired with an Orca-R2 digital camera (Hamamatsu) and either a Leica DMRXA2 microscope or a Zeiss Axioskop 2 plus microscope, driven by Volocity 6.3 software (Improvision, Perkin-Elmer). Images of pathology were analyzed semiquantitatively [13, 57] (Figures S1A–S1E). For pharynx, gonad and tumor pathologies, images were randomized, examined, assigned scores of 1-5 by two independent scorers, and mean values calculated and rounded. Here 1 = youthful, healthy appearance; 2 = subtle signs of deterioration; 3 = clearly discernible, mild pathology; 4 = well developed pathology; and 5 = tissue so deteriorated as to be barely recognizable (e.g., gonad completely disintegrated), or reaching a maximal level (e.g., large tumor filling the entire body width). Intestinal atrophy was quantified by measuring the intestinal width at a point posterior to the uterine tumors, subtracting the lumenal width and dividing by the body width. Yolk accumulation was measured by dividing the area of yolk pools with the area of the body visible in the field of view at 630x magnification.

#### Single Worm, Longitudinal Pathology Analysis

Worms were cultured individually at 20°C. On days 4, 7, 11, 14 and 18 of adulthood, each worm was imaged individually by Nomarski microscopy (Figures S1F and S1G). For imaging, microscope slides were prepared by taping two coverslips on the slide, at each edge, leaving an empty space in the middle for the agarose pad. The worm was then placed on a 2% agarose pad on the slide. Another coverslip was then placed on top, but resting on the two side coverslips, to reduce the pressure of the coverslip onto the worm. The slide was then placed on a PE120 Peltier cooling stage (Linkam Scientific) set to 4°C. Within minutes of cooling the nematodes ceased to move, and images were taken at 630x magnification using a Zeiss Axioskop microscope. After imaging, each worm was carefully recovered by pipetting 20 μL of M9 buffer between the top coverslip and the agar pad. The coverslip was then gently removed and the worm picked onto an NGM plate. Images of pharynxes, distal gonads, uterine tumors, PLPs and intestinal atrophy were analyzed as described above. The lifespan of each nematode was then measured.

#### Bodipy Staining

This was performed as described [18], except that worms were manipulated in 15 μL droplets held in parafilm micro-wells. Briefly, animals were washed 2x by transferring them successively into two droplets of M9 using a platinum wire pick. They were then transferred to a drop of 2% paraformaldehyde solution for 15-20 min and frozen/thawed 3x at −80°C/room temperature (RT). Animals were then carefully washed 3x by transferring them into successive M9 droplets. They were then transferred to 1 μg/mL BODIPY 493/503 (Invitrogen) in M9 solution for 1-2 hr at RT in darkness. The worms were finally washed 3x in M9 droplets and mounted for imaging (488 nm Exc./505-575 nm Em.) on a Zeiss LSM710 confocal microscope.

#### Lifespan Measurements

These were performed at 20°C or 25°C. Worms were either transferred daily during the reproductive period, or transferred at L4 stage to plates supplemented with 15 μM FUDR to block progeny production. Animals that died from internal hatching were censored. For *atg-13* RNAi lifespan trials, blind scoring was performed.

#### Generation of Transgenic Strains

Constructs were made using PCR fusion using primers as follows (a full list of primers is also provided in Table S2). To generate the *vit-6::mCherry* strain, where mCherry is fused to the C terminus of VIT-6, the 6,740 bp *vit-6* promoter and genomic region, excluding the stop codon, was amplified using F: 5′-TTCTTCTTTCGGTGGCTCTG-3′ and R: 5′-CTTCTTCACCCTTTGAGACCATATAGTCGAACTTGTCGCACT-3′. mCherry was amplified using F: 5′-ATGGTCTCAAAGGGTGAAGAAG-3′ and R: 5′-GATGGCGATCTGATGACAGC-3′. The 3′UTR was amplified using F: 5′-CTACCTCTTCTTCACAATCATACAC-3′ and R: 5′-ACTGTAGAAGTGAACTCTGTG-3′. The *vit-6* fragment was fused to mCherry using F: 5′-TGGAGACACAATAGAAGTCG-3′ and R: 5′-GTGTATGATTGTGAAGAAGAGGTAGCTACTTATACAATTCATCCATGCCAC-3′. The fused fragment was further fused with the 3′UTR using F: 5′-ATTCCACAGAAAGGATTGCAC-3′ and R: 5′-ATGCCGAGTTGTTTGAATTG-3′. For the *atg-13* intestinal rescue, the *ges-1* promoter was amplified using F: 5′-TTGTCTATTGGTATGGCTGC-3′ and R: 5′-GTACGTGTCGTACTCATTTACCATACAAGGAATATCCGCATCTG-3′. The *atg-13* 2,300 bp genomic region was amplified using F: 5′-ATGGTAAATGAGTACGACACGTAC-3′ and R: 5′-TGCAAGACTTCTGAGCAATG-3′. The two fragments were fused using primers F: 5′-GCGCTACCAATAAGGCTAAG-3′ and R: 5′-GAGCAATGTCGCAATGGAAAG-3′. *vit-6::mCherry* was microinjected at 1 μg/μL with 100 μg/μL *rol-6* coinjection marker to generate GA1711. GA1711 was crossed with GA1500 to obtain GA2100. VIT-6 is proteolytically cleaved, yielding YP115 and YP88 [58]. The VIT-6::mCherry fusion protein was ∼139 kDa in size (data not shown), consistent with YP115 being the C-terminal portion of VIT-6, as shown by protein sequence data obtained from YP115 [59]; i.e., mCherry is fused to YP115. *pges-1::atg-13* was injected into *atg-13(bp414)* at 80 μg/μL with 20 μg/μL *rol-6* coinjection marker. Two independent lines, GA1729 and GA1730, were used for experiments.

#### Electrophoretic Analysis of Yolk Proteins

Yolk protein levels were quantified by running worm protein extracts on PAGE gels and then staining with Coomassie blue dye as described [19]. 20 worms were picked into 25 μL of M9 buffer and frozen at −80°C. Samples where then thawed, and 25 μL of 2x Laemmli sample buffer (Sigma) added. Samples were incubated at 70°C, vortexed continuously for 15 min, incubated at 95°C for 5 min and spun at 6,000 rpm for 15 min. Samples were loaded onto Criterion XT precast gels 4%–12% Bis-Tris (Bio-Rad), using XT MOPS (Bio-Rad) as a running buffer, and stained and destained following standard protocols. Gels were analyzed using ImageQuant LAS-4000 (GE Healthcare). Protein band identification was based on published data [19, 21]. YP170, YP115 and YP88 were previously identified as vitellogenins by means of peptide mapping [60]. Correct identification in the present study of vitellogenins on protein gels was confirmed by the effect of *vit-5,6* RNAi, which abrogated YP170, YP115 and YP88 accumulation (Figure S4A). Yolk proteins were normalized to myosin and the ratio of actin to myosin was estimated to assess the reliability of myosin as a standard for normalization [19].

#### Yolk Protein Proportion Measurements

Two approaches were taken to estimate the proportion of total worm protein that is yolk protein: using densitometry or reference to protein standards of known concentration. For the densitometry-based approach, the density of individual vitellogenin bands (YP170, YP115 and YP88), and of the entire lane, of protein gels stained with Coomassie Blue were measured. The density of a whole lane (all protein) was set as 100%, and the percentages of the vitellogenin bands were calculated proportionally. The optimal threshold for densitometric reading was that which was just sufficient to exclude background from regions between gel lanes. For the protein standard-based approach, known amounts of protein standard were run alongside worm protein samples and gels stained with Coomassie Blue. The density of bands obtained from standard protein was used to construct a standard curve. The intensity of vitellogenin bands (YP170, YP115 and YP88) from the samples was then compared to the standard curve, to calculate the amounts of vitellogenin protein present. To estimate the proportion of yolk proteins to total protein, the estimated amount of yolk protein was compared with total protein content data obtained from protein quantification using BCA.

#### Total Nematode Protein Measurement

This was performed using bicinchoninic acid (BCA). Samples were prepared by adding 250 μL of CelLytic M buffer (Sigma) containing 1:1000 protease inhibitor cocktails. Samples were then mixed and sonicated using a Bioruptor (Cosmo Bio, Tokyo, Japan) for 8 min at 30 s intervals. Samples were centrifuged at 4°C at 6000 rpm for 15 min. The BCA method was performed in a 96-well plate, with each well containing 200 μL of testing solution and 25 μL sample or bovine serum albumin (BSA) standards. The plate was mixed gently and incubated at room temperature for 2 min and then incubated at 37°C for 30 min. Absorbance was then measured at 620 nm.

#### Confocal Microscopy

Bodipy stained animals were mounted on an 2.5% agarose pad between slide and coverslip in M9. 25-35 μm thick Z stacks were acquired every 0.75 μm through Zeiss Plan-Apochromat 40X or 63X 1.4 immersion lenses using Zeiss LSM710 (UCL) and LSM880 (LU) confocal microscopes equipped with 405 nm, 488 nm, 568 nm and 633 nm lasers, and controlled by the Zen software package. Bodipy, VIT-2::GFP, DHS-3::GFP and MANS-2::GFP were imaged using the 488 nm laser, while VIT-6::mCherry was imaged using the 568 nm laser. Confocal image analysis of Bodipy staining focused on the int1, 2 and 3 gut cells, averaging fluorescence from 7 consecutive Z-planes that passed through the intestinal lumen. Within the ROI, segmentation in intestinal and non-intestinal areas was performed plane by plane comparing brightfield and fluorescence images across the Z stack to delineate the intestinal limits. For DHS-3::GFP and MANS-2::GFP, scoring systems were established using a scale of 0 to 10 for DHS-3::GFP and 0 to 5 for MANS-2::GFP, with 0 representing no significant staining and 10 or 5 representing even and bright labeling. Intermediate scores correspond to various degrees of signal amount (area), density, heterogeneity and sharpness. The results shown are the average of two rounds of scoring for each dataset. Each confocal microscopy experiment combines 2 to 4 independent replicates of 5 to 30 worms for each genotype/time point combination. As fluorescence levels and dynamics vary between worms and slides, fluorescence intensity cannot be reliably related to lipid amounts, which is why lipid organelle area was chosen over fluorescence intensity as a quantitative measure. All fluorescence quantifications were performed on raw images while illustrations in supplemental figures were saturated to enable easy visualization.

#### Electron Microscopy

30-40 adult worms per condition were pre-fixed in a drop of 2% low-melting point agarose solution (kept below 30°C on a heat block) containing 2.5% glutaraldehyde, 1% paraformaldehyde in 0.1M sucrose, 0.05M cacodylate, and 0.02% levamisole. Worms were moved to a dissecting scope at room temperature and quickly aligned as the agarose set, and then cut in half using a razor blade. Half worms were realigned upon addition of another drop of melted agarose, and the gelled block was then trimmed before transferring into the fixative solution (2.5% glutaraldehyde, 1% paraformaldehyde in 0.1M sucrose, and 0.05M cacodylate) on ice. The agarose block was then stained and processed as described [61] (protocol 8), adjusting timings to account for slower diffusion through the agarose block. Serial 1 μm sections were taken for light microscopy inspection, and ultra-thin (70-80 nm) sections were cut at the region of interest using a diamond knife on a Reichert ultramicrotome. Sections were collected on slot grids and stained with lead citrate before viewing using a Joel 1010 transition electron microscope. Images were captured with a Gatan Orius camera and Gatan imaging software, and then exported in TIFF format.

#### Total Triacylglyceride Quantification

Biovision’s Triglyceride Quantification Kit (Mountain View, CA) was used to assay for triacylglyceride content. Animals were aged and counted before harvest. Samples contained approximately 300 animals in 100 μL Biovision assay buffer and were frozen in liquid nitrogen and thawed 100°C 3 times, followed by sonication to break the cuticle. Lipids were extracted in glass tubes with the Folch method and reconstituted in 100 μL assay buffer. Triacylglycerides were measured using the kit protocol.

#### Lipid Extraction and Mass Spectrometry Analysis

Lipidomic analysis was performed on about 3,000 to 10,000 worms per sample. Worms were counted and collected in M9 buffer. This was followed by washing: samples were centrifuged at 1000 rpm for 3min, to pellet the worms while leaving the bacteria in suspension. As much as possible of the supernatant was removed without disturbing the worm pellet and fresh M9 was then added. The process was repeated several times, until the supernatant was clear and free of bacteria. After a final wash in PBS, the worm pellet was resuspended in 1mL PBS and frozen at −80°C. The samples were thawed while vortexing at room temperature and transferred to 10mL silanized glass tubes using silanized glass pipettes. 1mL methanol and 2mL chloroform were added to each sample and vortexed to mix. 40 μL of lipid standard mixture (12:0/12:0/12:0-triacylglycerol [TG; 800 ng]) was used to spike each sample. After thorough mixing, samples were subjected to a modified Folch extraction. The lipid extracts were dissolved in 150 μL chloroform in silanised glass sample vials. 7 μL of sample was injected for positive ion lipids analysis and another 7 μL was injected for negative ion lipids analysis. In brief, different classes of lipids were separated on a normal phase Type C silica gel column (150 × 2.1mm, 4 μm, 100Å, MicoSolv Technology) with hexane/dichloromethane/chloroform/methanol/acetanitrile/water/ethylamine solvent gradient elution based on the polarity of head group using a Shimadzu Prominence HPLC system. Individual lipid species were identified and semi-quantified with high resolution (240 k at m/z 400) accurate mass analysis (mass accuracy < 5ppm) on a Thermo Orbitrap Elite mass spectrometer.

#### Intestinal Autophagy Assays

These were performed using strain DLM3 *ttTi5605 II; unc-119(ed3) III; uwaEx2 [vha-6p::CERULEAN-VENUS::lgg-1 + unc-119(+)]*, as described [26]. Animals were maintained on *E. coli* HT115 and the test group subjected to *atg-13* RNAi from L4 onward (20°C). This strain has intestine-limited expression of dFP (dimer) which contains two fluorescent protein-tagged monomers of the LGG-1 autophagosomal membrane protein. Upon autophagosome-lysosome fusion dFP is cleaved into mFP (monomer); thus, mFP/dFP ratio provides a relative measure of autolysosome formation.

#### Mating and Male Scent Exposure Tests

To test effects of mating on pathology, single hermaphrodites were mated with 3 males. On day 3, males were removed and mated hermaphrodites subsequently identified by the presence of male progeny, and then maintained for pathology analysis. To test effects of male scent, 60mm NGM plates were conditioned with males for 2 days. Males were then removed and hermaphrodites added. Plates were either conditioned with 150 males and then 30 hermaphrodites added, or with 60 males and then 30 hermaphrodites added. In a further protocol, 35mm NGM plates were conditioned with 30 males to which 30 hermaphrodites were added.

### Quantification and Statistical Analysis

#### Pathology Measurements

The Student’s t test was used. ANOVA and two-way ANOVA with Bonferroni correction were applied to take into account multiple comparisons. Correlations from single worm, longitudinal pathology analysis were analyzed using the Spearman Rank test and linear regression analysis. Benjamini-Hochberg corrections were used for multiple comparisons.

#### Survival Analysis

Statistical significance was estimated using log rank and Wilcoxon statistical tests executed using JMP 11 software (Data S3). Unless stated otherwise, three independent trials with N > 10 in each trial were used. All graphs display mean values and all error bars depict standard error of the mean (SEM).

## Supplemental Information

Document S1. Figures S1–S7 and Tables S1 and S2

Data S1. Analysis of Age Changes in Lipid Composition in Wild-Type *C. elegans*, Related to Figure 2

Data S2. Analysis of Age Changes in Lipid Composition in Fertilization-Defective *C. elegans rrf-3(bn26)* Mutants, Related to Figure 2

Data S3. Summary Statistics of Lifespan Trials, Related to Figure 6

Document S2. Article plus Supplemental Information
