# *C. elegans* Eats Its Own Intestine to Make Yolk Leading to Multiple Senescent Pathologies

**DOI:** 10.1016/j.cub.2018.10.003

**Published:** 2018-10-22

**Authors:** Marina Ezcurra, Alexandre Benedetto, Thanet Sornda, Ann F. Gilliat, Catherine Au, Qifeng Zhang, Sophie van Schelt, Alexandra L. Petrache, Hongyuan Wang, Yila de la Guardia, Shoshana Bar-Nun, Eleanor Tyler, Michael J. Wakelam, David Gems

(Current Biology *28*, 2544–2556.e1–e5; August 20, 2018)

In the original version of this article, the y axis in Figure 3A was mistakenly labeled as “mg/worm,” but it should have been “μg/worm.” This has now been corrected in the article online, and the corrected Figure 3A is also shown below. The authors apologize for this error and any confusion that may have resulted.

**Figure 3A:**
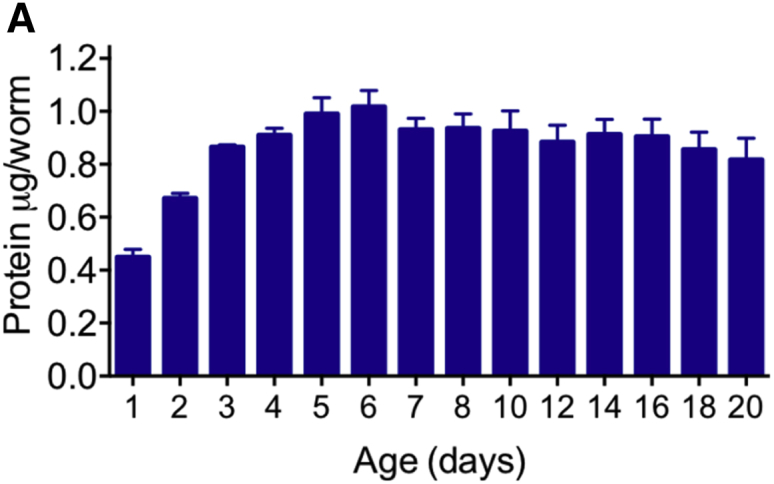
Evidence of Conversion of Intestinal Biomass into Yolk (corrected)

**Figure 3A:**
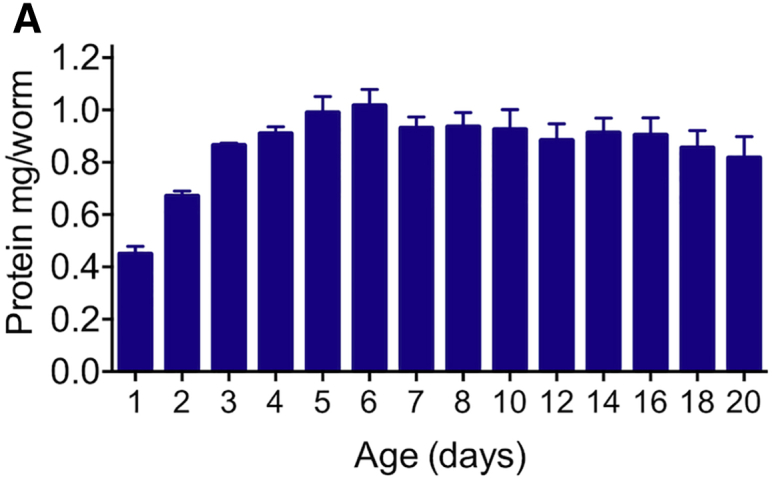
Evidence of Conversion of Intestinal Biomass into Yolk (original)

