# Supplementary material for: C. elegans Eats Its Own Intestine to Make Yolk Leading to Multiple Senescent Pathologies
Source: Curr Biol. 2018 Aug 20;28(16):2544–2556.e5. doi: 10.1016/j.cub.2018.06.035 (PMC6108400; doi:10.1016/j.cub.2018.06.035)
Supplement: Document S1. Figures S1–S7 and Tables S1 and S2 [file mmc1.pdf]

Current Biology, Volume *28*

Supplemental Information

C. elegans Eats Its Own Intestine to Make Yolk Leading to Multiple Senescent Pathologies

Marina Ezcurra, Alexandre Benedetto, Thanet Sornda, Ann F. Gilliat, Catherine Au, Qifeng Zhang, Sophie van Schelt, Alexandra L. Petrache, Hongyuan Wang, Yila de la Guardia, Shoshana Bar-Nun, Eleanor Tyler, Michael J. Wakelam, and David Gems

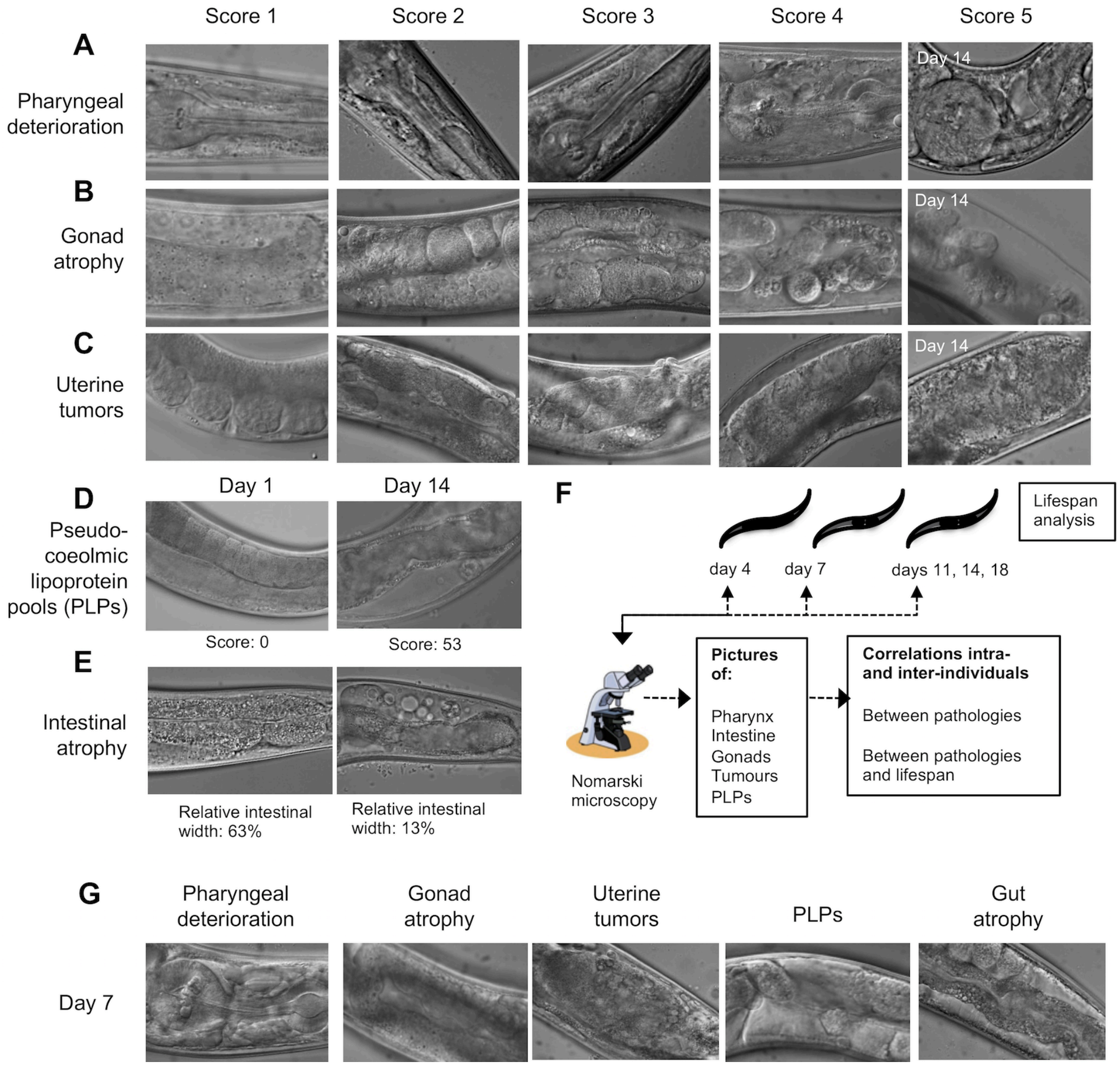

Figure S1. Measurement of severity of senescent pathology. Related to Figure 1. (A-C) For pharynx (A), gonad (B) and uterine tumor (C) pathologies, images were randomized, and given scores of 1-5. Here 1 = a youthful, healthy appearance; 2 = showing subtle signs of deterioration; 3 = clearly discernible, low level pathology; 4 = well developed pathology; and 5 = tissue so deteriorated as to be barely recognizable (e.g. gonad completely disintegrated), or reaching a maximal level (e.g. large tumor filling the entire body diameter). (D,E) Pseudocoelomic lipoprotein pool (PLP) formation (yolk accumulation) (D) and intestinal atrophy (E) were measured by dividing the total area of yolk pools with the area of the body visible in the field of view. (F) Experimental approach for measuring correlation between severity of senescent pathologies and lifespan, by combining serial imaging of pathology in individual nematodes, and then measuring their lifespan. (G) Examples of images of pathology at one of the time points examined (day 7).

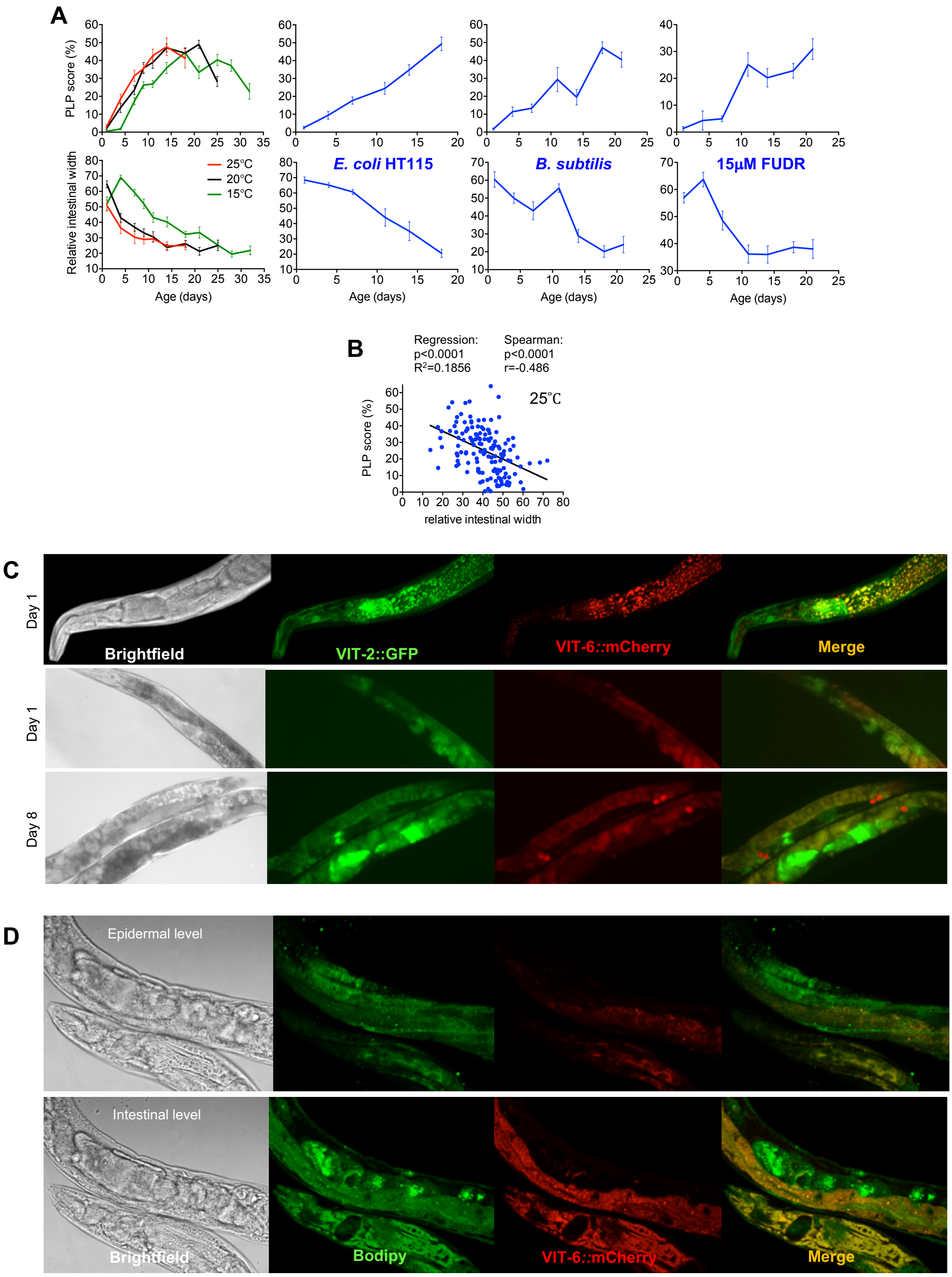

Figure S2. Senescent pseudocoelomic lipoprotein pool accumulation and intestinal atrophy. Related to Figure 2. (A) Co-occurrence of PLP accumulation (upper panels) and intestinal atrophy (lower panels) with age occurs at all temperature tested (far left), on *E. coli* K12 diets (center-left), on a *Bacillus subtilis* diet (center-right), and in presence of FUDR (far right). (B) PLP accumulation score and intestinal atrophy correlate at the individual worm level at 25˚C with FUDR. (C) Columns from left to right: brightfield, VIT-2::GFP, VIT-6::mCherry, VIT-2::GFP + VIT-6::mCherry merged. Day 1 adults show VIT-2::GFP and VIT-6::mCherry in the intestine (200X magnification) (top). Day 1 adults show VIT-2::GFP and VIT-6::mCherry in the intestine and embryos (100X) (middle). Day 8 adults show VIT-2::GFP and VIT-6::mCherry in the intestinal droplets and PLPs (100X) (bottom). VIT-2::GFP was also observed in the tumors and VIT-6::mCherry in the coelomocytes. (D) Columns from left to right: brightfield, Bodipy, VIT-6::mCherry, and Bodipy + VIT-6::mCherry merged. Day 10 adults, epidermal level, show Bodipy staining of lipid droplets within muscles (200X) (top). Day 10 adults, intestinal level, show yolky lipid within and outside of the intestine (200X) (bottom).

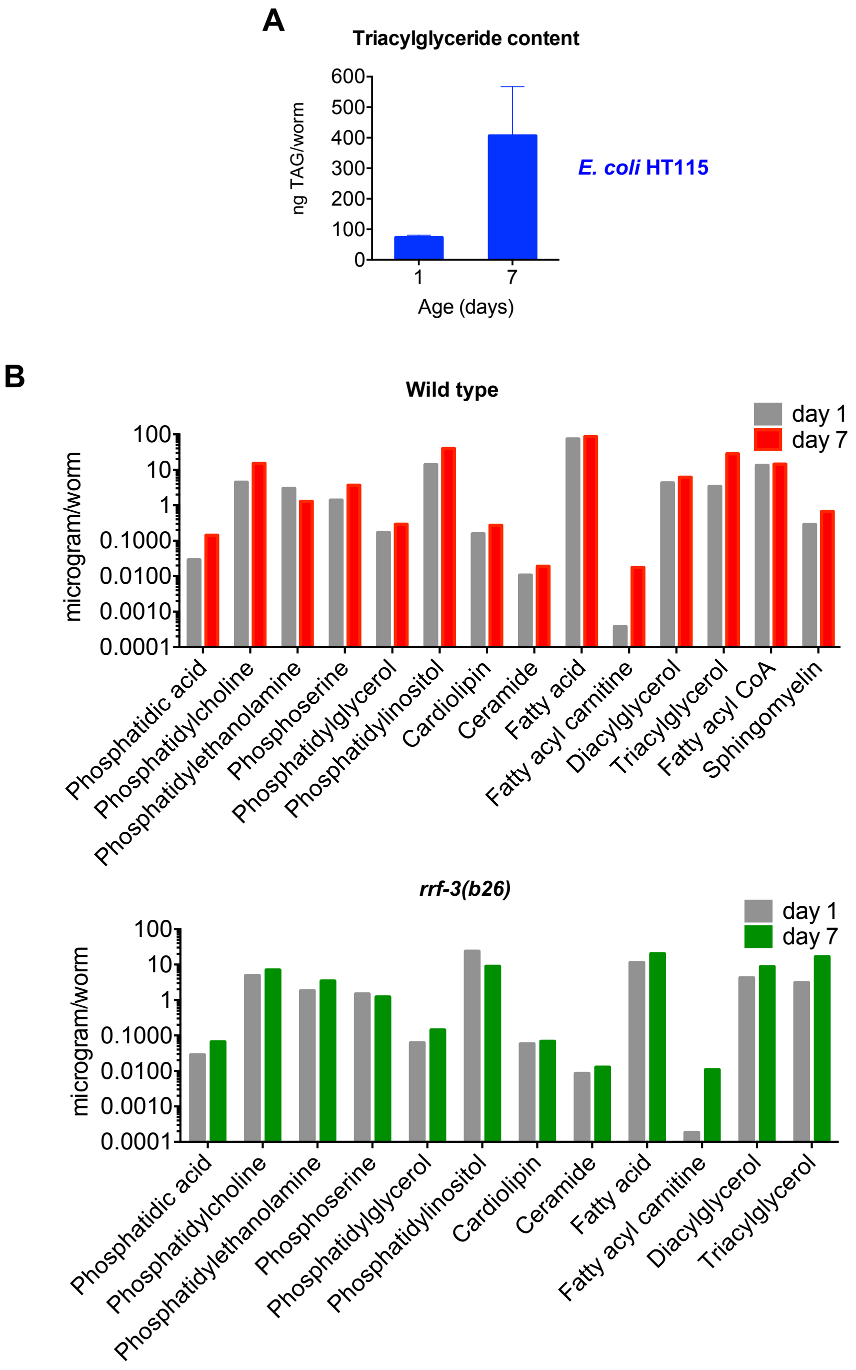

Figure S3. Alterations in lipid content. Related to Figure 2. (A) Quantification of total triacylglyceride (TAG) content, which increases 5-fold between day 1 and day 7 in wild-type hermaphrodites maintained on *E. coli* HT115. (B) Mass spectroscopy analysis of age changes in lipid profiles. In wild-type at 20˚C TAG content increases 8.2 fold (top). In sterile *rrf-3(b26)* mutants at 25˚C TAG content increases 5.0 fold (bottom).

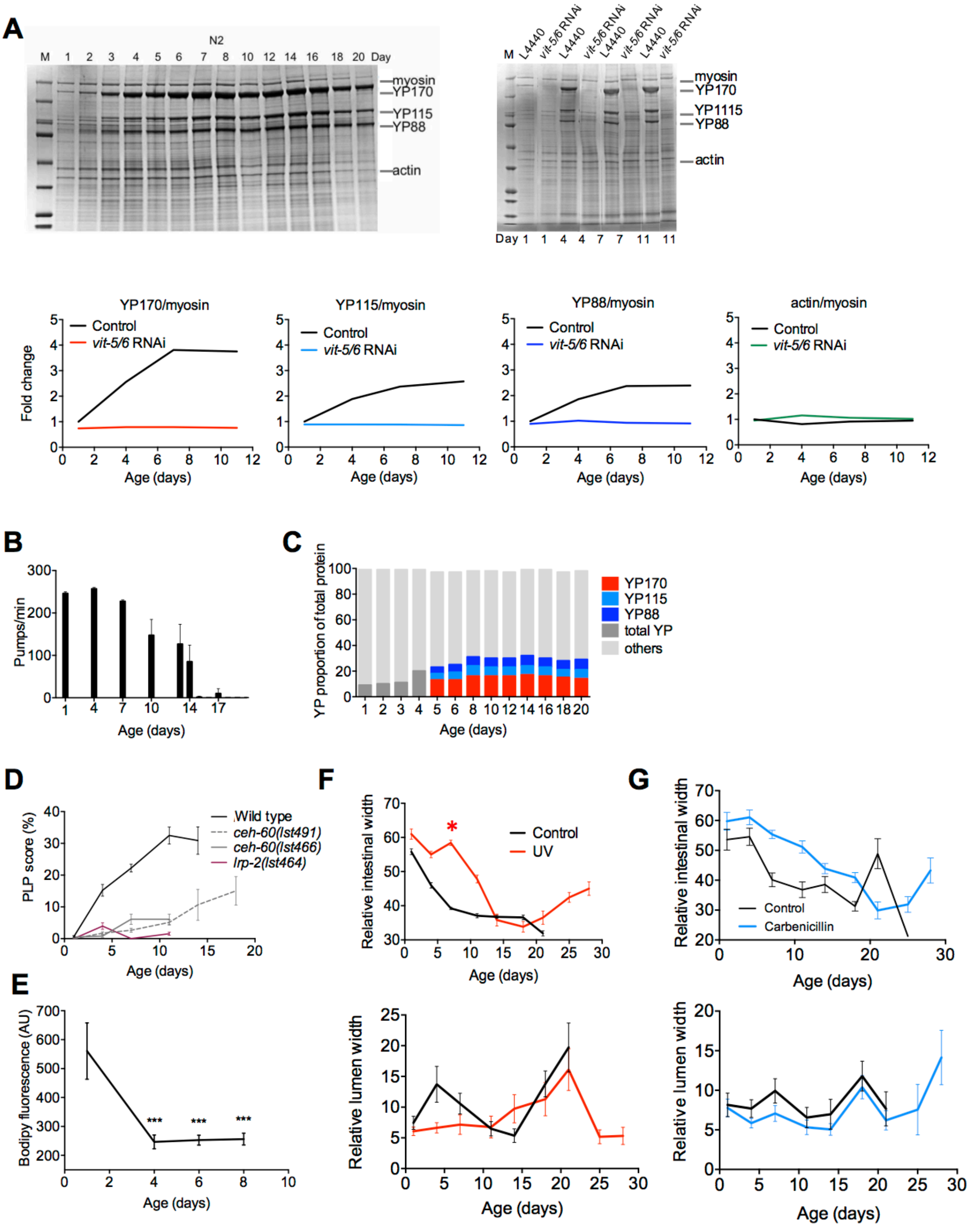

Figure S4. Vitellogenin accumulation and intestinal atrophy in aging hermaphrodites. Related to Figure 3. (A) Representative Coomassie stained gels showing the age-increase in yolk protein (YP) (vitellogenin) levels in N2 hermaphrodites (top left), and prevention of YP170, YP115 and YP88 accumulation after *vit-5,6* RNAi (top right, bottom; note that the original gel included 4 samples for each day, 2 of which were subsequently cropped from the image, since they showed other RNAi tests that are not relevant here). (B,C) Vitellogenin accumulation continues after reproduction despite a declining feeding rate. (B) Age decline in pharyngeal pumping rate. (C) Quantification of YP proportion of total protein using protein standards. According to this method, YP protein proportion increases from <10% on day 1 to ~35% on day 8. (D) Reduced accumulation of PLPs in *ceh-60* and *lrp-2* mutants. (E) Decrease with age in intestinal lipid content, measured with the fluorescent stain Bodipy. Trials conducted at 25˚C with 15µM FUDR. (F,G) Effects on intestinal atrophy of blocking bacterial proliferation with UV irradiation (F) or 4mM carbenicillin (G). Both treatments cause a delay but not a reduction in intestinal atrophy.

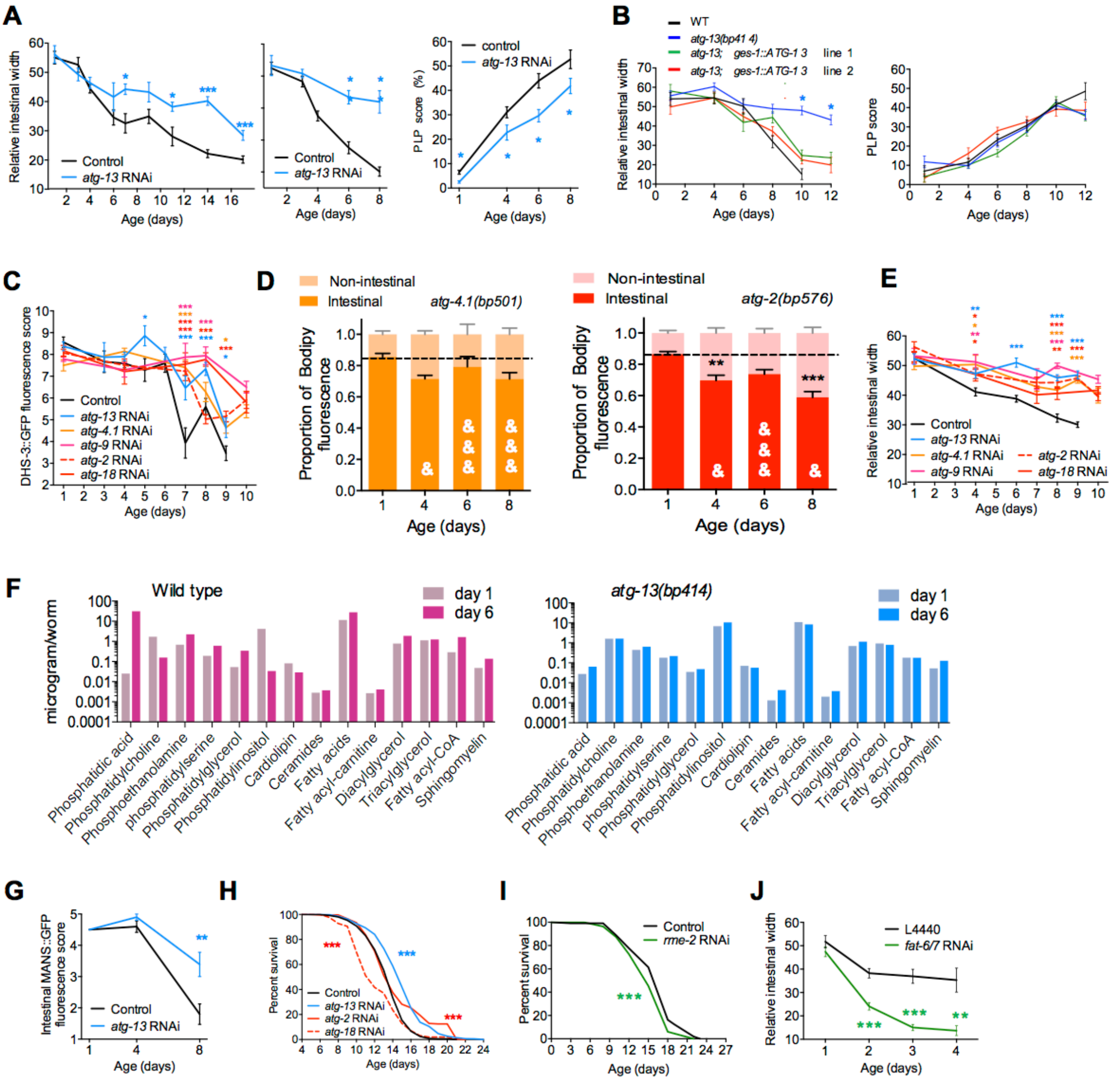

Figure S5. Inhibition of autophagy reduces visceral pathologies in aging hermaphrodites. Related to Figure 4. (A) Adult-limited *atg-13* RNAi suppresses intestinal atrophy (left, center) and PLP accumulation (right). The decline in relative intestinal width is reduced at 20˚C, no FUDR (left), and at 25˚C, 15µM FUDR (center). Suppression of PLP accumulation, 25˚C, 15µM FUDR (right). (B) Intestine-specific rescue of *atg-13(bp414)* restores intestinal atrophy to wild-type levels, 25˚C, no FUDR (left) though not PLP accumulation (right). (C) Adult-limited inhibition of autophagy delays DHS-3::GFP-tagged intestinal lipid droplet reduction, *N*=2 trials. (D) *atg-4.1(bp501)* (left) and *atg-2(bp576)* (right) (autophagy defective) retard senescent redistribution of lipids (Bodipy staining); ** *p*<0.01, *** *p*<0.001, comparison to day 1; & *p*<0.05, &&& *p*<0.001, comparison to age-matched control (shown in Figure G3). (E) Adult-limited inhibition of autophagy delays intestinal atrophy, *N*=2 trials. F) *atg-13(bp414)* reduces the age-associated lipidomic shift occurring between day 1 and day 6 of adulthood; 25˚C, 15µm FUDR. (G) Quantification of Golgi abundance using MANS::GFP. Age-related decrease in Golgi abundance is suppressed by *atg-13* RNAi. (H) Adult-limited *atg-2* RNAi or *atg-13* RNAi increase while *atg-18* RNAi decreases lifespan; 25˚C, 15µm FUDR. (I) Adult-limited *rme-2* RNAi decreases lifespan; 25˚C. (J) *fat-6/7* RNAi increases intestinal atrophy, *N*=2 trials.

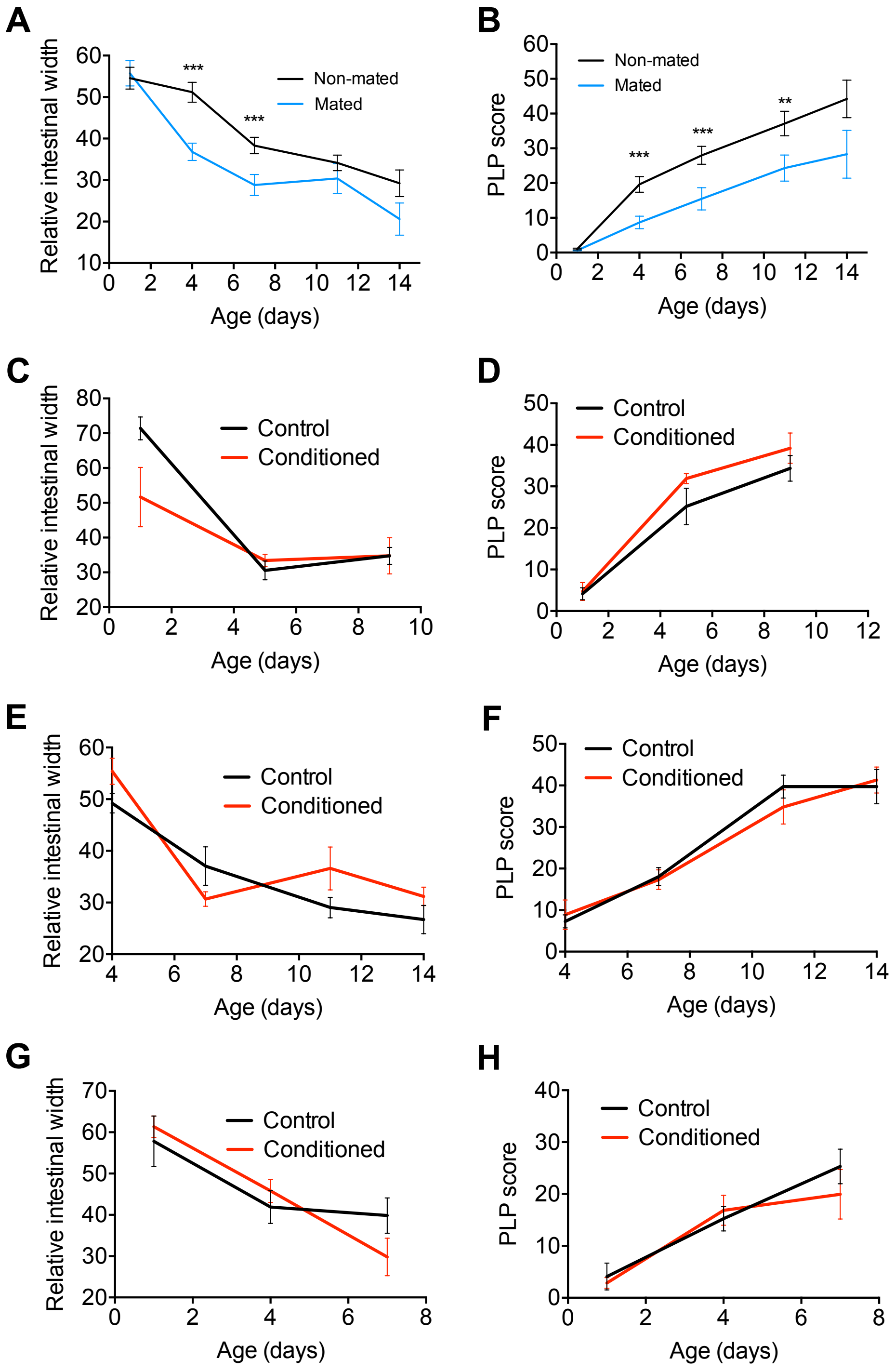

Figure S6. Effects of mating and exposure to males on visceral pathology. Related to Figure 6. (A,B) Effects of mating on intestinal pathology (A) and PLP accumulation (B). Each hermaphrodite was mated with 3 males for 3 days. (C-H) Effects of exposure to male scent on intestinal pathology and PLP accumulation. Conditioning experiments were performed with different ratios of conditioning males and assayed hermaphrodites. (C,D) 150 males:30 hermaphrodites. (E,F) 30 males:30 hermaphrodites. (G,H) 60 males:30 hermaphrodites (35mm plates).

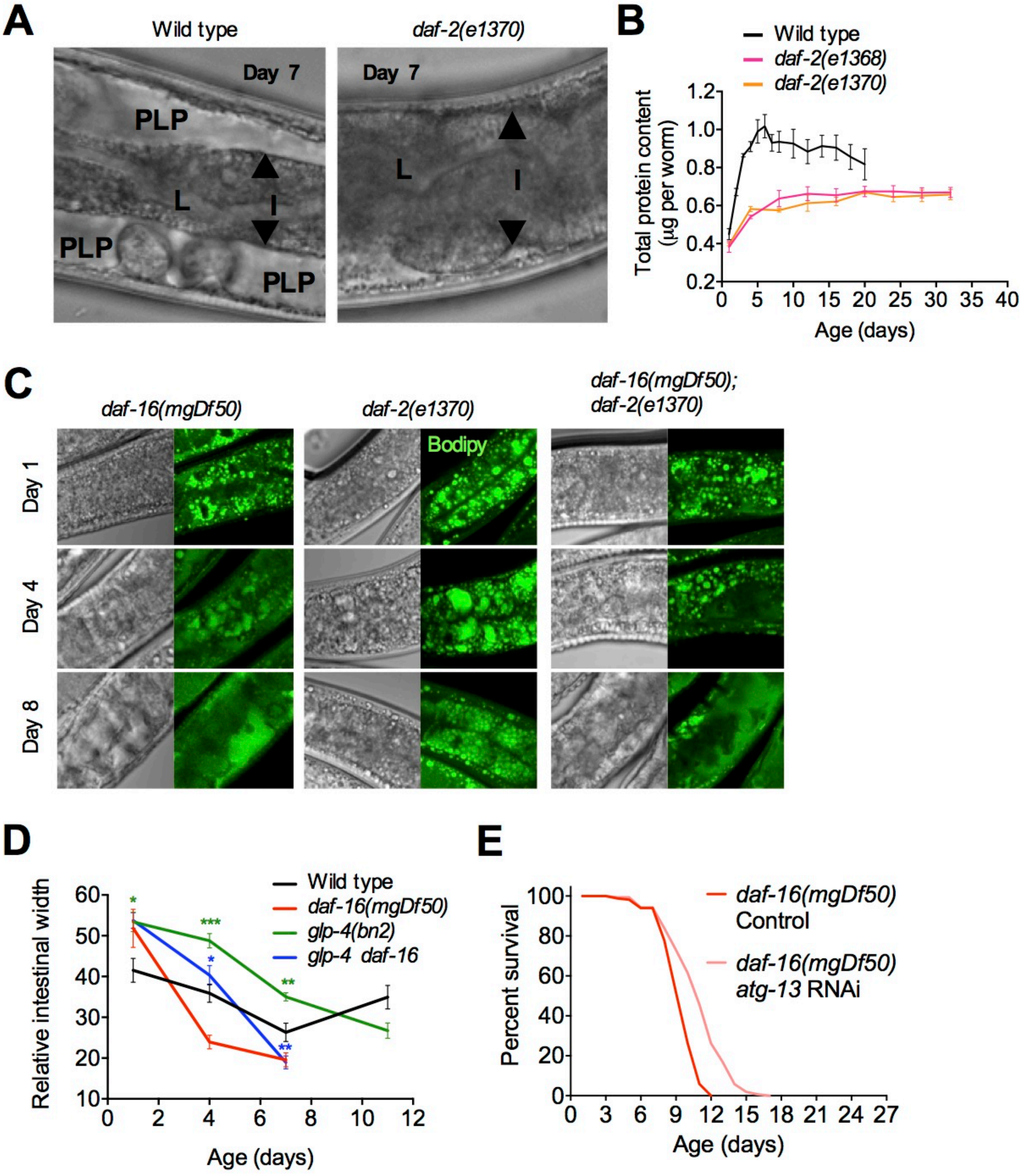

Figure S7. Gut-to-yolk biomass conversion results in gut atrophy and PLP accumulation. Related to Figure 7. (A) Left: Nomarski image of an N2 hermaphrodite mid-body at day 14 of adulthood (20˚C), showing atrophied intestine and PLPs. Right: age-matched *daf-2(e1370)* mutant mid-body. Note full-sized intestine and absence of PLPs (representative images). (B) Comparison of total protein contents during aging in age-matched wild type, *daf-2(e1368)* and *daf-2(e1370)* mutants. *daf-2* mutant protein content increases much less with age than wild type. (C) Representative pictures of gentle-fix Bodipy staining in the anterior intestine and surrounding tissues in *daf-16(mgDf50)* (left), *daf-2(e1370)* (middle), and *daf-16; daf-2* mutants (right), illustrating DAF-16-dependent preservation of intestinal fat stores and prevention of fat transfer in *daf-2* mutants at days 1, 4 and 8 of adulthood at 25˚C. (D) Blocking germline growth using *glp-4(bn2)* delays intestinal atrophy, and this effect is largely *daf-16* dependent, 25˚C, *N*=2 trials. Statistical comparisons, green: *glp-4 vs.* wild type; blue, *glp-4 daf-16 vs. glp-4*.

Lipid species (ng per worm)	20˚C no FUDR				25˚C 15μM FUDR			
	**Wild type day 1**	**Wild type day 7**	***rrf-3* day 1**	***rrf-3* day 7**	**Wild type day 1**	**Wild type day 6**	***atg-13* day 1**	***atg-13* day 6**
Phosphatidic acid	0.0197	0.1060	0.0287	0.0664	0.0254	30.4577	0.0280	0.0639
Phosphatidylcholine	1.8859	6.0791	4.9495	7.0140	1.7199	0.1552	1.6067	1.6504
Phosphoethanolamine	1.1001	1.7826	1.8407	3.4344	0.6872	2.2187	0.4427	0.6395
Phosphatidylserine	0.2397	0.8127	1.4856	1.2290	0.1925	0.5967	0.1815	0.2185
Phosphatidylglycerol	0.1081	0.1725	0.0629	0.1434	0.0526	0.3416	0.0349	0.0488
Phosphatidylinositol	9.5943	30.0363	23.8472	8.9514	4.2035	0.0330	6.8700	10.6244
Cardiolipin	0.1187	0.1681	0.0589	0.0686	0.0807	0.0287	0.0708	0.0578
Ceramides	0.0012	0.0029	0.0085	0.0128	0.0029	0.0036	0.0013	0.0044
Fatty acids	64.78	75.82	11.45	20.19	11.61	27.41	10.91	8.43
Fatty acyl-carnitine	0.0002	0.0131	0.0002	0.0109	0.0027	0.0040	0.0021	0.0039
Diacylglycerol	2.7751	4.5304	4.2648	8.7420	0.7787	1.8314	0.6915	1.1561
Triacylglycerol	1.5930	13.2132	3.0936	16.7488	1.1455	1.2188	0.9313	0.8023
Fatty acyl-CoA	13.479	14.470	-	-	0.290	1.599	0.183	0.181
Sphingomyelin	0.0637	0.2101	-	-	0.0480	0.1352	0.0536	0.1271
Others	4.858	12.967	12.477	5.577	1.448	5.007	2.197	2.254
TOTAL	100.62	160.39	63.56	72.19	22.28	71.04	24.21	26.26

"-" indicates missing values (compromised samples).

Table S1. **Lipid profiles of young and middle-aged *C. elegans* hermaphrodites. Related to Figure 5.** Genotypes tested are wild type (N2), *rrf-3(b26)* (temperature-sensitive sterile) and *atg-13(bp414)*.

Primer target DNA	Direction	5' to 3' primer sequence
vit-6 (promoter +CDS)	Forward	TTCTTCTTTCGGTGGCTCTG
vit-6 (promoter +CDS)	Reverse	CTTCTTCACCCTTTGAGACCATATAGTCGAACTTGTCGCACT
mCherry	Forward	ATGGTCTCAAAGGGTGAAGAAG
mCherry	Reverse	GATGGCGATCTGATGACAGC
3'UTR	Forward	CTACCTCTTCTTCACAATCATACAC
3'UTR	Reverse	ACTGTAGAAGTGAACTCTGTG
vit-6::mCherry fusion	Forward	TGGAGACACAATAGAAGTCG
vit-6::mCherry fusion	Reverse	GTGTATGATTGTGAAGAAGAGGTAGCTACTTATACAATTCATCCATGCCAC
vit-6::mCherry::3'UTR fusion	Forward	ATTCCACAGAAAGGATTGCAC
vit-6::mCherry::3'UTR fusion	Reverse	ATGCCGAGTTGTTTGAATTG
ges-1 (promoter)	Forward	TTGTCTATTGGTATGGCTGC
ges-1 (promoter)	Reverse	GTACGTGTCGTACTCATTTACCATACAAGGAATATCCGCATCTG
atg-13 (CDS)	Forward	ATGGTAAATGAGTACGACACGTAC
atg-13 (CDS)	Reverse	TGCAAGACTTCTGAGCAATG
ges-1p::atg-13 fusion	Forward	GCGCTACCAATAAGGCTAAG
ges-1p::atg-13 fusion	Reverse	GAGCAATGTCGCAATGGAAAG

Table S2. Oligonucleotides used to produce PCR fusions for transgenic strain generation. Related to STAR Methods. The promoter of *vit-6* and coding genomic sequence were fused to *mCherry* coding sequence and *vit-6* 3'UTR in 5 sequential PCR reactions (10 first primers). *atg-13* genomic coding sequence was fused to the *ges-1* promoter for the gut-specific rescue of ATG-13 expression in *atg-13(bp414)* mutants (6 last primers).
